# Transient Cholesterol Effects on Nicotinic Acetylcholine Receptor Cell-Surface Mobility

**DOI:** 10.1371/journal.pone.0100346

**Published:** 2014-06-27

**Authors:** Gonzalo Almarza, Francisco Sánchez, Francisco J. Barrantes

**Affiliations:** Laboratory of Molecular Neurobiology, Biomedical Research Institute, Pontifical Catholic University of Argentina (UCA) and National Scientific and Technical Research Council of Argentina (CONICET), Buenos Aires, Argentina; Weizmann Institute of Science, Israel

## Abstract

To what extent do cholesterol-rich lipid platforms modulate the supramolecular organization of the nicotinic acetylcholine receptor (AChR)? To address this question, the dynamics of AChR particles at high density and its cholesterol dependence at the surface of mammalian cells were studied by combining total internal reflection fluorescence microscopy and single-particle tracking. AChR particles tagged with a monovalent ligand, fluorescent α-bungarotoxin (αBTX), exhibited two mobile pools: i) a highly mobile one undergoing simple Brownian motion (16%) and ii) one with restricted motion (∼50%), the rest being relatively immobile (∼44%). Depletion of membrane cholesterol by methyl-α-cyclodextrin increased the fraction of the first pool to 22% and 33% after 15 and 40 min, respectively; the pool undergoing restricted motion diminished from 50% to 44% and 37%, respectively. Monoclonal antibody binding results in AChR crosslinking-internalization after 2 h; here, antibody binding immobilized within minutes ∼20% of the totally mobile AChR. This proportion dramatically increased upon cholesterol depletion, especially during the initial 10 min (83.3%). Thus, antibody crosslinking and cholesterol depletion exhibited a mutually synergistic effect, increasing the average lifetime of cell-surface AChRs∼10 s to ∼20 s. The instantaneous (microscopic) diffusion coefficient *D*_2–4_ of the AChR obtained from the MSD analysis diminished from ∼0.001 µm^2^ s^−1^ to ∼0.0001–0.00033 µm^2^ s^−1^ upon cholesterol depletion, ∼30% of all particles falling into the stationary mode. Thus, muscle-type AChR exhibits heterogeneous motional regimes at the cell surface, modulated by the combination of intrinsic (its supramolecular organization) and extrinsic (membrane cholesterol content) factors.

## Introduction

In brain synapses, variations in the number of neurotransmitter receptors and the time they spend at the surface membrane directly affect synaptic efficacy and plasticity, that is, long-term potentiation (LTP), long-term depression (LTD) and other biologically important synaptic phenomena. Likewise, e.g. at the peripheral cholinergic synapse, the number of nicotinic acetylcholine receptor (AChR) molecules at the surface membrane plays a major role in synaptic functionality.

The AChR is the prototype of the family of Cys-loop receptors within the superfamily of rapid ligand-gated ion channels. In adult myotubes, this protein is highly concentrated in a small area juxtaposed and restricted to the nerve terminal, patched at the extraordinary density of 10,000–20,000 particles/µm^2^, its density falling sharply in the rest of the plasma membrane to less than <100 particles/µm^2^ refs [1], [2]. The functional efficacy of this peripheral synapse, as well as other synapses, heavily depends on its strength. This in turn is directly related to the number of receptors present at the synapse, which depends on the equilibrium between two sets of factors: i) lateral diffusion into and out of the synaptic region from non-synaptic (“extrasynaptic”) areas, and ii) the trafficking and turnover of receptors at the cell surface, determined by the rate and extent of biosynthesis and exocytic delivery to the plasmalemma on the one hand, and removal of surface receptors by internalization (endocytosis) on the other.

Cholesterol is an abundant component in the postsynaptic membrane [3] and it has been demonstrated that this lipid affects the functional properties and distribution of the AChR [4]. Marsh and Barrantes [5] demonstrated the lateral heterogeneity of lipids in the postsynaptic membranes of *Torpedo* electrocyte: protein-associated lipids are immobilized with respect to bulk membrane lipid, and subsequent work has shown that cholesterol-like molecules form part of this protein-immobilized pool [4], [6], [7]. The presence of cholesterol is also important for maintaining agonist-dependent functional states of the AChR [8]. It has been proposed that there are two cholesterol populations in AChR-rich membranes from *Torpedo*: an easily extractable fraction that influences the bulk fluidity of the membrane and a tightly bound receptor-associated fraction [9]. The lipid “raft” hypothesis proposes that specific lipid species self-associate to form microdomains or platforms that can intervene in protein partition, signaling and other functional properties [10], . There is also evidence that AChRs interact with cholesterol-rich lipid domains *in* *vitro* and *in* *vivo* [12]–[17]. In our laboratory it has been demonstrated that cholesterol plays a key role in the trafficking of the AChR along the early secretory [18] and endocytic [19] pathways and also affects AChR distribution in the plasma membrane [19], [20].

The pioneer study of Axelrod et al. [21] using the fluorescence recovery after photobleaching (FRAP) technique demonstrated that in developing muscle cells the highly clustered AChRs present in large (20–60 µm) patches are practically immobile, with an effective lateral diffusion coefficient (*D*) of <10^−12^ cm^2^ s^−1^ (<10^−4^ µm^2^ s^−1^). The translational mobility of diffusely distributed AChRs in other regions of the same plasma membrane is slightly faster (*D* ∼0.5×10^−2^ µm^2^ s^−1^). In central nervous system synapses, the rapid lateral exchange of surface receptors with those in non-synaptic areas is thought to underlie the plastic behavior of excitatory receptors (for a review see Choquet and Triller [22]). Nicotinic receptors in brain also appear to follow similar principles [23], [24]. In one of these studies [24] the lateral mobility of neuronal-type α7 AChRs in chick ciliary ganglion neurons, measured using quantum dots, was reported to be 0.070 µm^2^ s^−1^ and 0.188 µm^2^ s^−1^ in synaptic and non-synaptic regions, respectively; disruption of lipid rafts by methyl-β-cyclodextrin treatment increased the mobility of α7 AChRs but not that of α3 AChRs, concluding from these and other data that AChR mobility is receptor-subtype specific.

In the present work we studied the dynamics of the α2*δ*ε-type adult muscle AChR at the plasma membrane of CHO-K1/A5 cells tagged with a monovalent ligand (fluorescent α-bungarotoxin) or a polyvalent ligand (monoclonal antibody). Antibody crosslinking notably reduced AChR nanocluster lateral mobility, increasing the average lifetime of the aggregates and reducing their average displacement. Synergistic effects were observed between antibody-mediated crosslinking and cholesterol levels on the dynamics of the receptor at the plasmalemma.

## Materials and Methods

### Materials

Alexa-Fluor-labeled α-bungarotoxin (Alexa^488^-αBTX) and Alexa^488^-labeled anti-rat IgG secondary antibody were purchased from Molecular Probes, Eugene, OR. Methyl-β-cyclodextrin (CDx) was purchased from Sigma Chem. Co. (St. Louis, MO). The monoclonal mAb 210 antibody against the main immunogenic region of the α-subunit was a gift from Dr. Jon Lindstrom, Univ. of Pensylvannia, Philadelphia.

### Cell culture

CHO-K1/A5 cells were grown in Ham’s F12 medium supplemented with 10% fetal bovine serum (BFS) for 2–3 days at 37°C before experiments as in Roccamo et al. [25].

### Acute cholesterol depletion/enrichment of cultured cells

Acute cholesterol depletion prior to fluorescent labeling was accomplished by treating CHO-K1/A5 cells with 10–15 mM CDx in Medium 1 (“M1”: 140 mM NaCl, 1 mM CaCl_2_, 1 mM MgCl_2_ and 5 mM KCl in 20 mM HEPES buffer, pH 7.4) essentially as in Borroni et al. [19] except that incubation proceeded for 20 minutes at 37°C.

### Labeling of cells with fluorescent probes

In order to label plasma membrane AChR, CHO-K1/A5 cells were stained with Alexa^488^-αBTX at a final concentration of 1 µM for 1 hour at 4°C in M1, washed, and mounted for microscopy. Crosslinking of plasma membrane AChR in CHO-K1/A5 cells was accomplished by labeling with mAb210 monoclonal antibody for 1 hour at 4°C in M1, washing, and labeling with Alexa^488^-labeled rat anti-mouse antibody for 1 h at 4°C in M1, followed by washing and final mounting for microscopy.

### Total internal reflection fluorescence (TIRF) microscopy

Measurements were made on a Nikon TE-2000-U inverted fluorescence microscope (Microlat, Nikon Argentina). The excitation source was a 50 mW 488 nm Ar laser (Lasos AG, Germany). The laser light was passed through a laser cleanup filter (Z488/10X with 10 nm band pass, Chroma Technology Corp., Bellows Fall, VT), reflected by a dichroic mirror (Z488RDC back coated laser dichroic mirror, with roughly 515 nm long pass, Chroma) and through emission filter HQ525/50 (Chroma), and focused through a Nikon planapochromatic TIRF 100x, 1.49 N.A. oil immersion objective onto the sample. The excitation intensity was varied potentiometrically via the laser power supply unit or by inserting neutral density filters in the beam path, and was measured at the microscope objective with a power meter. Thermal control of the samples was accomplished using a Bionomic thermoelectrically controlled chamber model BC-250 (20/20 Technology, Inc., Wilmington, NC). Individual images were acquired with a Quantum-EM-512 16-bit electron-multiplying CCD camera (Photometrics, Tucson, AZ) with a 1.0x projection lens, yielding a pixel size of 160 nm in the image plane of the camera. Image acquisition for movies was performed with 100 ms exposure in streaming mode using the software SlideBook (Intelligent Imaging Innovations, Boulder, CO) and exported as 16 or 8-bit images. Subsequent analysis was carried out using Image J (NIH, USA, http://rsb.info.nih.gov/ij/) or Matlab (MathWorks, see below) and further exported to Excel (Microsoft) for further data processing (see below).

### Single-particle tracking (SPT) analysis

Initial SPT of TIRF imaging was carried out using routines in the SlideBook acquisition and analysis software. First, the center position and intensity of each particle above an intensity threshold value in each frame were established and particles were linked together into trajectories. To maximize the fidelity of tracking, parameters were chosen using low intensity thresholds for detection to ensure oversampling (i.e. a significant amount of spurious detection was permitted in each frame) rather than undersampling (i.e., loss of legitimate trajectories). Batch analysis was subsequently undertaken using the software developed by Jaqaman et al. [26], currently under the name U-track, in a Matlab R2008b (The MathWorks Inc., Boston, MA) environment. Given a set of detected particles throughout a time-lapse images sequence, the algorithm first links the detected particles between consecutive frames, and then links the detected particles track segments generated in the first step to simultaneously close gaps and capture particle merge and split events [26]. The initial particle assignment is temporally “greedy” [26], but the subsequent track segment assignment is accomplished via temporally global optimization, overcoming the shortcomings of algorithms relying solely on greedy assignment strategies. Both steps use global optimization in space. The method allows the robust tracking of particles under high density conditions such as those found in the case of AChR particles expressed at the surface of CHO-K1 cells. Codes were developed in Matlab to handle the statistical analysis of the data produced by the Jaqaman analysis. These codes generated meta-data on the number of particles, mean displacements, initial and final coordinates, mean velocities, average lifetime of a given trajectory and the corresponding graphical representations associated with such data.

### Ensemble analysis of AChR particle motions: Mean-squared displacement (MSD) analysis

Typically, a diffusion process in *d* dimensions is characterized by the ensemble-averaged, mean-squared displacement (MSD) [27]:
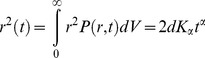
(1)assuming spherical symmetry and an isotropic environment, such that P(r, t) is the probability density to find the particle at (radial) distance r away from the origin at time t after release of the particle at r = 0 at time t = 0. In Eq. 1 above, the anomalous diffusion exponent is taken into account by introduction of the exponent α [27]. In the limit α = 1 simple Brownian diffusion results. Two forms of anomalous diffusion result from other values of α: subdiffusion in the case 0<α<1, and superdiffusion for α>1. Here we calculated the MSD of particles (nanometer-sized AChR supramolecular clusters [20]) diffusing in the 2-dimensional plane of the cell membrane.

MSD (*t*) analysis followed the general approach outlined by the classical work of Kusumi et al. [28] and references therein. For each trajectory of a particle in the plane of the membrane, (Δ*r*(Δ*t*))^2^, the 2-dimensional MSD was calculated for every time interval according to the formula [29], [30]:
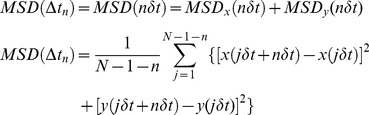
(2)

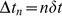
(3)with a variable Δ*t*, which depended on the acquisition frame rate of the streamed time-series, and where (x(*jδt*+n*δt*) and (j*δt*+n*δt*)) describe the particle position following a time interval *δt_n_* = n*δt* after starting at position (x(*jδt*), y(*jδt*)). *N* is the total number of frames in the stream recording sequence, and *n* and *j* are positive integers, with *n* determining the time increment [28]. We fitted the MSD data accordingly and interpreted the slope as the two-dimensional diffusion coefficient.

### Calculation of the diffusion coefficient, *D*

For 2-dimensional Brownian motion, the microscopic diffusion coefficient of a symmetric particle in an isotropic medium, *D*_2D_, is given by:
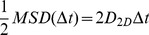
(4)


The 2-dimensional diffusion coefficient, *D*, was calculated by a linear fit of the MSD <r^2^> plot vs. time, according to the relation:
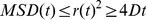
(5)using the definitions (1–3) above. Only the first few data points are normally used to calculate the microscopic *D*_2D_, because the stochastic nature of Brownian motion causes an error in measuring 2-dimensional MSD, so that *σ*_MSD_ increases with Δ*t* [30]:



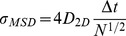
(6)In practice, *D* was determined by a linear fit of the MSD values at 2Δ*t*, 3Δ*t*, and 4Δ*t*, referred to as short-range diffusion coefficient or *D*_2–4_ [28]. *D*_2–4_ is convenient because it can be determined independently of the motional modes. On average, *D*_2–4_ is larger than the values of *D* determined using Eqs. 5–12 of Kusumi et al. [28] by a factor of 1.2 in the case of restricted diffusion. This case is precisely the one with the greatest deviation. Although the distribution of D_2–4_ is wider than that of D_0–4_, the advantages of this approach as applied to the analysis of actual experimental data have been pointed out by Saxton [31], [32]. Since the number of experimental points at the initial portion of the data is relatively scarce, it is useful to apply a minimal square method thus, obtaining an expression of D in terms of the mean square displacements (MSD) <r^2^(n)>. If all points are given equal weight, the slope of the minimal square line is given by:
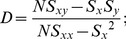
(7)where N is the number of experimental points, 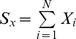. Assuming 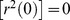 is an experimental point, one obtains:



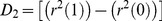
(8a)

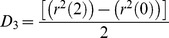
(8b)

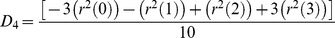
(8c)

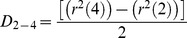
(8d)For measurement of the confinement radius, the MSD(Δ*t*) was averaged for the first ms (this depended on the acquisition rate used in each particular experiment) and the data were fitted to the following algorithm defining confined movement:
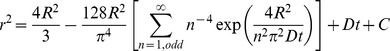
(9)where R is the confinement radius, *D* is the diffusion coefficient, 4*Dt* is free diffusion of the bounded object, and *C* is an offset constant [28]. Fitting of the experimental data was carried out by least-squares analysis using the Gauss-Newton procedure.

### Calculation of the relative displacement, *RD*

The curves of MSD-*Δt* show positive and negative displacements with respect to a straight line of slope 4D for the case of trajectories exhibiting directed and confined diffusion, respectively (see below). Large displacements imply a high probability that the trajectories depart from a simple Brownian motion. In order to quantify these different motional regimes, a relative displacement parameter was calculated according to the algorithm:
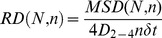
(10)where *MSD(N, n)* represents the MSD for the interval *nΔt* in a sequence of N images and *4D_2–4_ nΔt* is the expected mean value of the MSD for a particle moving under simple Brownian conditions with a diffusion coefficient *D_2–4_*. For simple Brownian trajectories the value of *RD(N, n)* should be unity.

### Characterization of motional regimes

Three motional regimes (simple Brownian, directed Brownian and confined or restricted motions) were first simulated using a custom-written routine in Matlab that generated initial conditions on the basis of random numbers (see Figures S2–S7). The simulated MSD vs. time plots clearly differentiated between three types of curves (Figure S8). Analysis of the motional modalities undergone by the actual AChR nanoparticles exploited the results of these simulations (Figure S8). Particle motional regimes were classified into the above three different types of motions. Trajectories with durations shorter than 15 frames (i.e. between 1.8 and 2.25 s) were categorized as “undetermined”. In order to characterize the motional regime for each individual trajectory, the MSD for different intervals was taken into account. The initial MSD points of those trajectories with durations >15 frames were fitted with linear and quadratic equations. The sum of the quadratic error was evaluated for each fit, and their errors compared (Figure S9). If the fits with the linear equation exhibited a low error, the trajectories were classified as corresponding to simple Brownian motion. If the quadratic approximation produced a better fit we then looked further into whether it followed an upward or a downward trend, corresponding to directed Brownian motion or restricted motion, respectively (Figures S8 and S9).

### Data meta-analysis: Cluster properties

The spatial arrangement of AChR particles in CHO-K1/A5 cells was analyzed using several statistical approaches. This required knowledge of the size, brightness, density and number of AChR particles in the TIRF images. Full width at half maximum (FWHM) was determined along the longitudinal (x) and the transversal (y) axis from the fitted 2D Lorentzian data using PSF v_05, a macro written in Matlab. The positional x, y coordinates of the centroids of the diffraction-limited AChR particles in each frame were localized through least squares fitting a two-dimensional Gaussian PSF using the QUICK-PALM plugin [33] in the Image-J platform or the software Localizer [34] written in IGOR PRO (WaveMetrics, Inc.). In the latter case, the clusters were segmented by a generalized likelihood ratio test algorithm [35] designed to detect PSF-shaped (i.e. Gaussian-like) spots. The number of trajectories typically analyzed was in the order of 800 (4%) and 700 (ca. 10%) out of a total of 15,000 and 8,000 for BTX and mAb-labeled samples, respectively. A symmetric Gaussian function was fitted to the spots using Levenberg–Marquardt least-squares minimization. Localizations that were too close together to be independent were discarded.

A graphical quantitative cluster analysis based on Ripley’s K function [36], [37] and a second-order neighborhood analysis of point patterns [38], as recently implemented by Owen and coworkers [39] was applied to 4.0×4.0 µm areas of cropped TIRF image stacks. Stacks varied between 80 and 1000 frames. The image plane pixel size was set at 0.16 µm (resulting from bead calibration of the microscope setup), with a FWHM of 0.48 µm. For the generation of the cluster maps, *L*(r) values at a spatial scale of 200 nm were computed for each point (*j*) individually as in [39] and [40]. Once each particle had been assigned a value for the above spatial scale, a quantitative pseudocolored cluster map was generated. Cluster statistics (number of clusters per cell surface area, number of particles within or outside the boundaries of the operationally defined cluster value, etc.) were calculated.

A further assessment of randomness or clustering of the particles was carried out by applying the paired correlation function, *G(r)*. *G(r)* is a measure of density correlations; it estimates the probability of finding a second particle at a distance *r* away from a given particle and compares the data to the expected values for a random distribution of particles. *G(r)* was calculated using the software SpPack [41]. *G(r)*s were computed from the ensemble positions by calculating the number of particles in a shell of radius r around each particle, normalized by the area of the shell, and corrected for edge effects. This measure was averaged over all the particles to obtain the final *G(r).* Values of *G(r)*>1 indicate non-random distribution, which can be assumed as particle clustering [42]–[44]. *G(r)* was also employed to determine the length scale of the clustering [45].

## Results

### Detection of fluorescent-labeled AChRs in living CHO-K1/A5 cells

Previous work from our laboratory showed that adult muscle-type AChR heterologously expressed at the cell surface of a mammalian clonal cell line, CHO-K1/A5, occurs in the form of fine diffraction-limited punctate particles of about 0.2 µm [19], which we will refer to simply as “particles” throughout. Subsequent work using superresolution microscopy -stimulated total emission depletion (STED)- disclosed that these diffraction-limited puncta corresponded to nanometer-sized clusters with an average diameter of about 50–100 nm upon crosslinking with monoclonal antibodies, which we have dubbed “nanoclusters” [20]. In the present work AChRs were labeled with Alexa^488^-α-bungarotoxin (αBTX), a fluorescent quasi-irreversible antagonist which tags membrane receptors in a stable manner [19], or with a primary anti-AChR monoclonal antibody (mAb210) followed by staining with Alexa^488^-labeled secondary antibody. We imaged AChR of CHO-K1/A5 cells under through-objective fluorescence TIRF microscopy at the maximal acquisition rate afforded by our imaging system, thus gaining enhanced contrast and temporal resolution at the expense of spatial resolution in order to characterize the dynamics of these receptor particles in the living cell.

**Figure 1:**
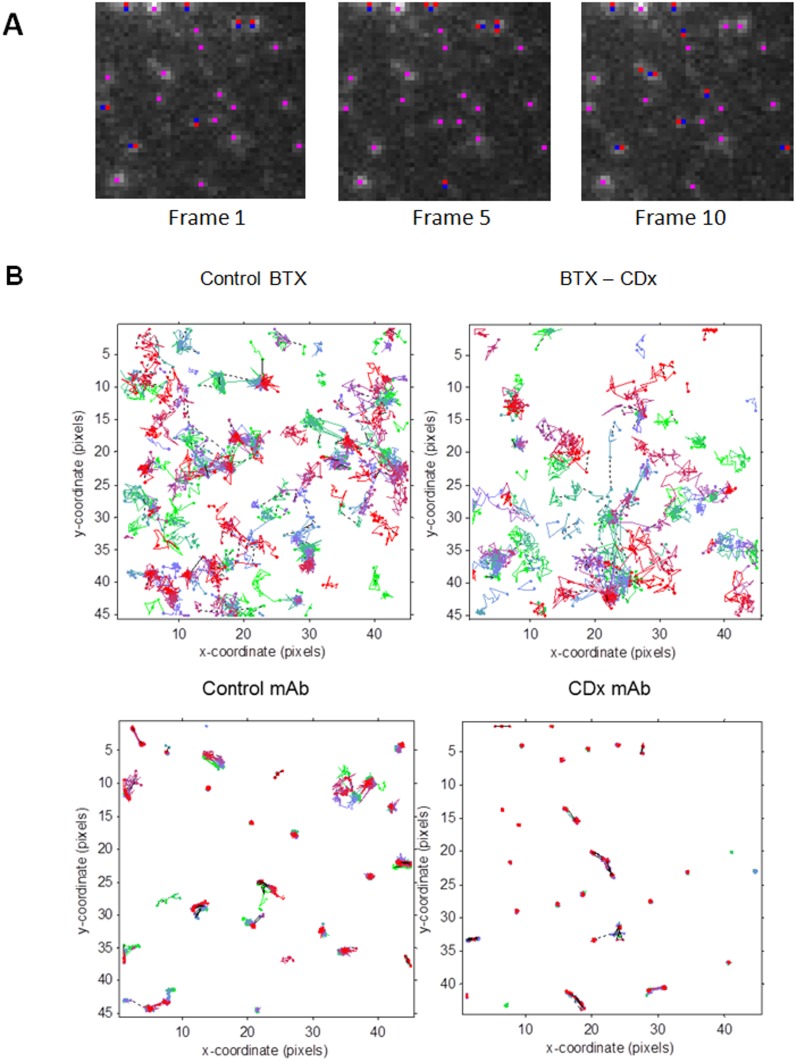
Detection and visualization of individual particles in a fluorescence TIRF time-series. A) The three images correspond to the initial frames (1, 5 and 10, respectively) of a time-series obtained from a 7.2×7.2 µm region of a CHO-K1/A5 cell treated with 10 mM CDx for 20 min and labeled with Alexa^488^-αBTX. AChR particles labeled in blue correspond to those detected in the initial phase using the U-track method of Jaqaman et al. [26]. Pink-labeled pixels correspond to coincidences between initial estimation of a detected particle and the same, when validated upon optimization by application of the algorithms of Jaqaman et al. [26]. B) Visualization of the trajectories followed by several cell-surface AChR particles. The two upper figures correspond to CHO-K1/A5 cells labeled with a monovalent ligand (AlexaFluor^488^α-BTX, left) or BTX followed by CDx treatment. The two lower figures correspond to cells labeled with a multivalent ligand (monoclonal anti-AChR mAb210 antibody followed by AlexaFluor^488^-conjugated IgG secondary antibody) at 4°C and recorded as in Fig. 1. The different trajectories are color-coded to facilitate their identification and their temporal scale: initial (green), middle (blue) and final (red) portions of the trajectory are shown in each case. As a rule, particles were followed for periods of ∼25–40 s (300 initial steps at 12.4–7.5 Hz, 80–130 ms/frame). Notice the relative immobility of the mAb210 antibody-labeled samples in comparison to the α-BTX-labeled samples. Analyzed using the U-track method of Jaqaman et al. [26].

**Figure 2:**
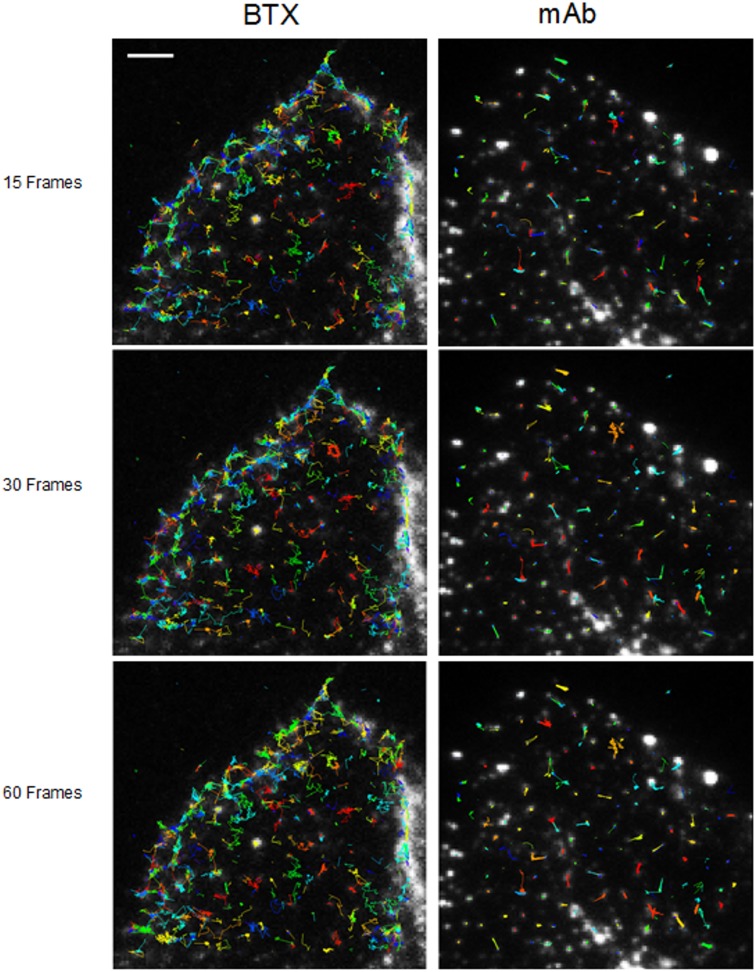
Multiple trajectories of AChR particles labeled with BTX and mAb210, respectively. Sequence of 15 successive frames (out of a total of 1024) corresponding to control BTX- (left column) and mAb (right column)-labeled samples superimposed on the raw TIRF initial frames. Particles were initially localized using a fixed-width Gaussian fitting. Detected particles were subsequently analyzed for their trajectories with the software Localizer [34] ran in an Igor-Pro environment. Typical total number of trajectories was in the order of 800 (4%) and 700 (ca. 10%) out of a total of 15,000 and 8,000 for BTX and mAb-labeled samples, respectively. Scale bar = 3 µm.

**Figure 3:**
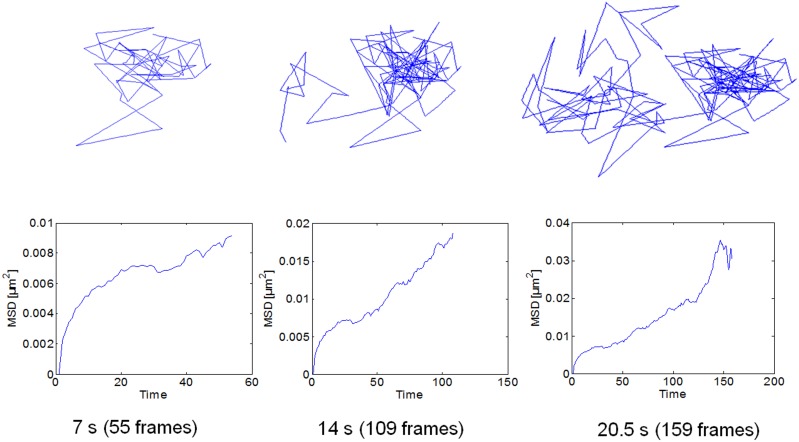
Individual trajectory and mean-squared displacement (MSD) of an AChR particle. The upper row shows the displacement of the same particle at the different time points indicated in the graph. The MSD (*t*) of the fluorescent-labeled particle was calculated for the initial 15 time lag intervals, Δ*t*, for trajectories longer than a critical number (15) of frames. In the example shown, the time-series was acquired at 20°C in a control CHO-K1/A5 cell labeled with Alexa^488^-α-BTX for 1 h at 4°C. The 2-dimensional MSD (*t*) for each particlés trajectory (Δ*r*(Δ*t*))^2^, was calculated for every time interval [28]–[30].

**Figure 4:**
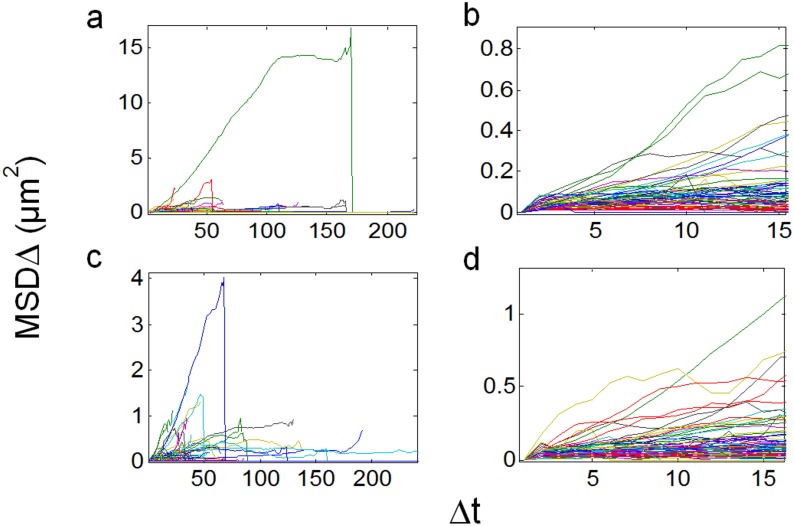
MSDs of particle trajectories in fluorescent BTX-labeled samples. Raw traces and b) MSD vs. *Δt* for *all* trajectories having more than 15 frames, including outliers; b) zoom of (a) showing the 15 initial *Δt* intervals only. Both a and b are control fluorescent BTX-labeled samples, and c–d are the corresponding samples treated with 10 mM CDx. MSD is expressed in µm^2^ and Δt in number of frames.

**Figure 5:**
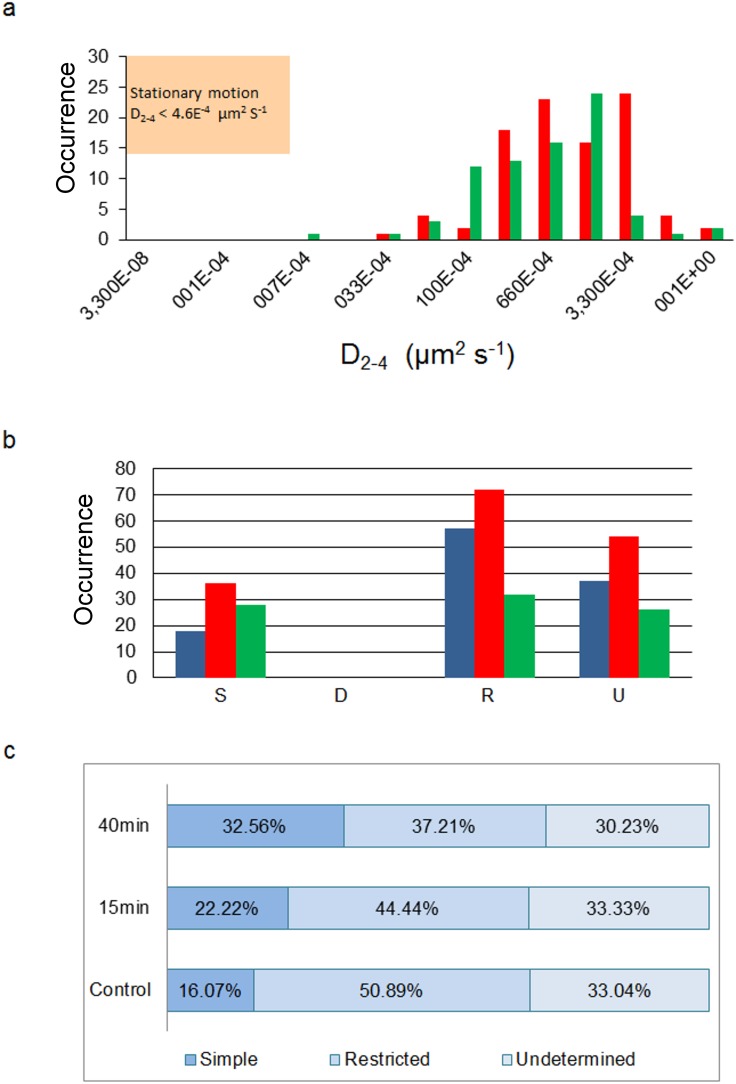
Motional regimes and diffusion coefficient of fluorescent BTX-labeled AChR particles. a) Distribution of diffusion coefficients for all trajectories for control fluorescent BTX-labeled samples (red) and samples treated with 10 mM CDx (green), respectively. The shaded inset (top left corner, same applies to Fig. 7a) indicates the upper limit of the microscopic diffusion coefficient D_2–4_, which in turn determines whether a trajectory can be classified as stationary or not. A stationary trajectory can be considered a special case of the Brownian simple and restricted motions, in which the particle does not diffuse beyond a critical value (4.6×10^−12^ cm^2^ s^−1^ [28]). As is apparent in Figure 7e, very few particles fall within this category, with little if any mobility. b) Proportion of different types of motion undergone by all trajectories (S-simple; D-directed; R-restricted or confined; U-undetermined). The color code represents different experiments: control fluorescent BTX-labeled samples (blue), samples treated with 10 mM CDx for 15 min (red) and for 40 min (green), respectively. c) Representative percentages of types of motion corresponding to the histograms in Figure 5b.

Static views -single time frames- of live control () or cholesterol-depleted (Figure S1b) CHO-K1/A5 cells labeled with Alexa^488^-αBTX and imaged in the TIRF mode under 488 nm laser excitation exemplify image planes corresponding to the coverglass-adhered cell surface. The basal membranes exhibit in both cases diffraction-limited particles of about 0.2 µm. The complete time series, acquired in the streaming mode to maximize temporal resolution, typically consisted of 1024 frames. Some of these puncta exhibited little if any mobility, remaining for several hundred frames in the same coordinates, whereas other AChR nanoclusters lasted for only a few frames (kymograph in Figure S1c, arrows). The density of these puncta is high, yet there is enough contrast and their separation suffices to track the trajectories of such mobile particles for the duration of several hundred frames (at frame rates between 80 and 133 ms/frame) (see Figs. 1–5 and statistics in Table 1). In the case of the more densely populated (in terms of AChR particles) control samples, an average of 110–120 compound tracks involving between 3,700 and 7,700 particles could be followed (Table 1); as previously observed in fixed specimens using wide-field [19] or superresolution [20] microscopy, live cholesterol-depleted cells characteristically showed a lesser number of AChR particles, and an average of roughly half the number of particles than that of control samples could be followed as a function of time, involving in both cases mobile populations having path lengths of a few (2–5) µm (see Table 1).

**Table 1:** Mobility parameters of AChR particles in samples labeled with Alexa^488^α-BTX or with a primary anti-AChR monoclonal antibody (mAb210) followed by staining with Alexa^488^-labeled secondary antibody, with or without treatment with 15 mM methyl-β-cyclodextrin (CDx).

Experiment	Average lifetime (s)	Average displacement(µm)	Average velocity(µm/ms)	Total No.of particles(in all frames)	Total No.compound tracksanalyzed
BTX control	4.06±0.78	4.05±0.27	0.0011±0.0002	4535	121
BTX CDx(10 min)		4.54±0.36	0.0010±0.0002	3759	101
BTX CDx(15 min)	5.30±0.80	4.42±0.06	0.0009±0.0001	4574	128
mAb control	10.47±0.31^a^	4.36±0.02^a^	0.0004±0.0001	7772	69
mAb CDx(10 min)	11.06±3.11^b^	2.13±0.25^b^*	0.0002±0.0001	5987	53
mAb CDx(20 min)	13.41±1.44^c^	4.76±0.72^c^	0.0005±0.0001	3755	41
mAb CDx(40 min)	19.96±0.68^d^*	5.70±1.18^d^	0.0003±0.0001	4409	29
Average lifetime:					
Average displacement:					

Using the strategy of Jaqaman et al. [26] all particles contained within sub-regions of CHO-K1/A5 cells (usually a 7.2×7.2 µm ROI) were detected in time-series for total durations of ∼25–40 s (i.e. using the initial ∼300 frames). Figure 1 shows an example of the first step -detection- of individual particles from a CHO-K1/A5 cell treated with 10 mM CDx for 20 min and labeled with Alexa^488^-αBTX.

### Dynamic tracking of cell-surface AChR nanoclusters in living CHO-K1/A5 cells; immobilization by antibody crosslinking

Figure 2 shows the trajectories followed by AChR particles at the cell surface resulting from the second step of the Jaqaman et al. [26] analysis and rendered using the software Localizer. CHO-K1/A5 cells were labeled with a monovalent ligand (AlexaFluor^488^α-BTX, *upper panel*) or a multivalent ligand (anti-AChR mAb210 monoclonal antibody followed by AlexaFluor^488^-conjugated IgG secondary antibody, *upper panel*) at 4°C. The average total number of trajectories was twice as high (∼120) in control CHO-K1/A5 cells labeled with the monovalent ligand (AlexaFluor^488^α-BTX) as in those labeled with the monoclonal antibody. Most dramatic was the relative increase in average lifetime of the mAb210 antibody-labeled samples in comparison to the α-BTX-labeled samples (Table 1), apparent just by simple inspection of the particle walks, and corroborated by the reduction of the diffusion of particles in the former case (Figure 2 and see below).

### Mean-squared displacement (MSD) of individual trajectories: heterogeneity of motional regimes

MSD analysis of individual trajectory data was carried out next for all particles satisfying the criteria imposed previously in order to obtain information on the motional characteristics of the observed AChR particles. The MSD of the fluorescent-labeled particles was calculated for the initial 15 time lag intervals, Δ*t*, for trajectories longer than a critical number of frames, using Eqs. 1–3 in Material and Methods. A typical trajectory of a fluorescent (AlexaFluor^488^-αBTX)-labeled particle undergoing Brownian diffusion in a confined cell-surface area (see below) is shown in Figure 3 (*upper panel*). The particle, followed for several seconds, corresponds to a control CHO-K1/A5 cell at 20°C. The resulting MSDs at increasing intervals are shown in each case (Figure 3, *lower panel*).

**Figure 6:**
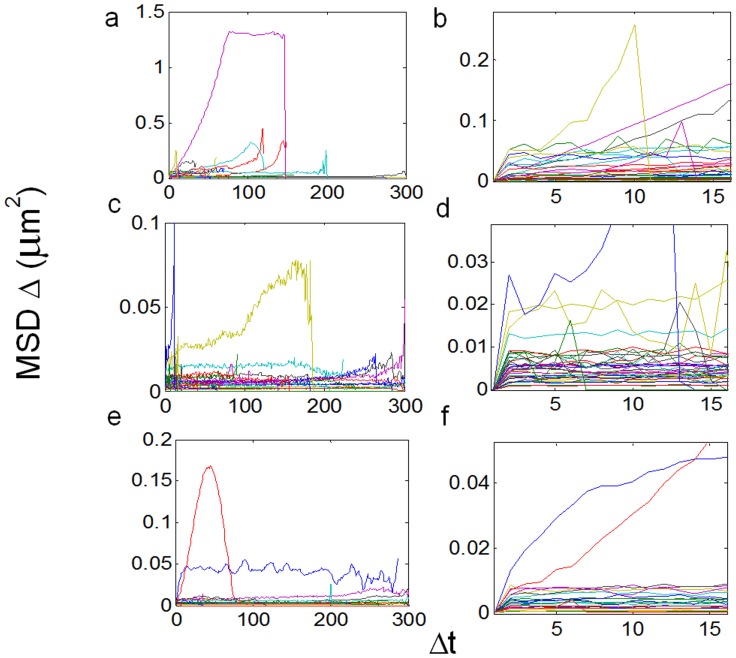
MSDs of particle trajectories in mAb210 antibody-labeled samples. A) Raw traces and b) MSD vs. *Δt* for all trajectories having more than 15 frames, including outliers, as in Fig. 5; b) Zoom of (a) showing the 15 initial *Δt* intervals only. Both a and b correspond to the control fluorescent mAb-labeled samples, and c–d and e–f the corresponding samples treated with 10 mM CDx (20 and 40 min exposure, respectively). MSD is expressed in µm^2^ and Δt in number of frames.

When the above type of analysis was extended to all particles satisfying the eligibility criteria (as detailed under Material and Methods) it was possible to gain information on the type of mobility exhibited by AChR particles. Examples of MSD vs. *Δt* for all trajectories having more than 15 frames are shown in Figures 4 and 6 for CHO-K1/A5 cells labeled with AlexaFluor^488^α-BTX or with mAb210 antibody followed by AlexaFluor^488^-conjugated IgG secondary antibody, respectively. The microscopic diffusion coefficient, *D*_2–4_, was calculated using these data and Eqs. 4–8 (Material and Methods). The distribution of diffusion coefficients for all trajectories is plotted in Figures 5a and 6a for BTX and mAb, respectively. In the case of CHOK1/A5 cells labeled with the monovalent ligand, α-BTX, one can see that the microscopic (short-range [28]) diffusion coefficient, *D*_2–4_, shifted from a wide distribution spanning from ∼6.7×10^−4^−1 µm^2^ s^−1^ (∼6.7×10^−12^−1×10^−8^ cm^2^ s^−1^) to a much narrower distribution with an upper limit close to 5×10^−4^ µm^2^ s^−1^ (Figure 5a) upon cholesterol depletion. No particles were seen to fall within the region established for immobile particles (“stationary” regime). In the case of the antibody-labeled samples, the proportion of slow-moving particles was significantly higher, with a net displacement of particle motion towards the immobile region (Figure 6a). *D*_2–4_ values as low as ∼3.3×10^−5^ µm^2^ s^−1^ (lower limit) to ∼6.7×10^−2^ µm^2^ s^−1^ (upper limit) were observed (Figure 6a). In the case of BTX-labeled AChR particles, no trajectories were observed within the category of stationary motion for control or CDx-treated samples (Figure 6a). In the case of samples labeled with antibodies the situation was different. Control samples labeled with mAb210 already exhibited a substantial proportion (19.4%) of immobilized particles. This proportion dramatically increased upon cholesterol depletion of the cells, especially during the initial 10 min (83.3%). Interestingly, this short exposure to CDx appears to suffice to alter the mobility properties of monoliganded (BTX, Figures 4 and 6) and mAb-crosslinked AChRs (Figure 6). The percentage of stationary particles fell to 57.1% and 26.7% after 20 min and 40 min treatment with CDx, respectively (Figure 6a). Recently Simonson and coworkers [46] have reported a 2-dimensional diffusion coefficient of 0.1 µm^−2^ s^−1^ for α7-5HT3 chimaeric AChRs heterologously expressed in HEK cells.

**Figure 7:**
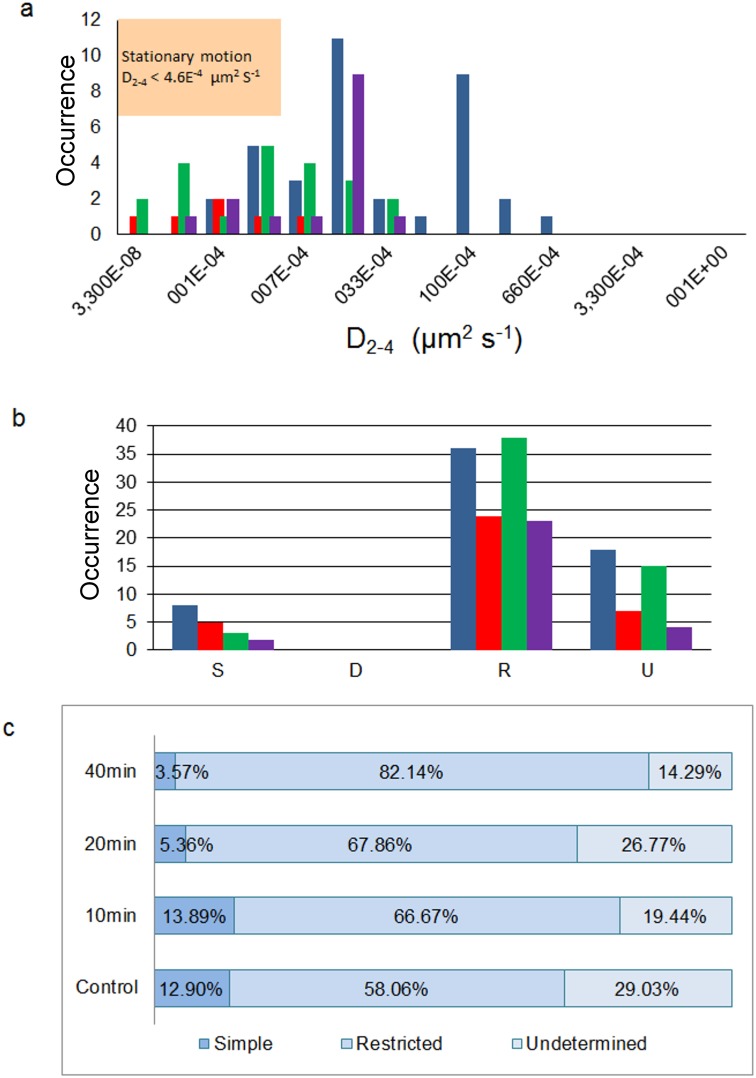
AChR particle mobility is drastically hindered by antibody crosslinking. a) Histogram depicting the distribution of diffusion coefficients for all trajectories for control fluorescent mAb-labeled samples (blue), and for samples treated with 10 mM CDx for 10 min (red), 20 min (green) and 40 min (purple), respectively. The shaded inset indicates the upper limit of the microscopic diffusion coefficient D_2–4._ One can clearly observe that the amount of particles with D_2–4_ below the critical value is higher than in the case of the BTX-labeled samples (Figure 5a). b) Histogram showing the proportion of different types of motion undergone by all trajectories (S-simple; D-directed; R-restricted or confined; U-undetermined). The color scale codes for control fluorescent mAb-labeled samples (blue), and samples treated with 10 mM CDx for 10 min (red), 20 min (green) and for 40 min (purple), respectively. c) Representative percentages of types of motion corresponding to the histograms in Figure 7b.

From the above analyses it was possible to calculate the different types of motion undergone by all trajectories, which fell essentially into simple Brownian and restricted (confined) diffusion. A variable proportion of particles’ trajectories with durations shorter than 15 frames (i.e. between 1.8 and 2.25 s) were categorized as “undetermined” in each experimental case (Figures 5b and 7b). In the case of BTX-labeled particles, the majority of the particles under all experimental conditions (control, CDx 15 min and 40 min, respectively) exhibited restricted motions or confined motions within a region. Some of the trajectories were not sufficiently long and were therefore classified as “undetermined”, with a lesser number of particles falling within the simple Brownian category. In no case was directed motion observed (Figure 5b). The different types of motion undergone by AChR nanoparticles in BTX-labeled samples is shown in detail in Figure 5c. In the case of antibody-labeled samples the majority of the particles exhibited restricted motion, with very few showing simple Brownian motion. This is made very apparent in the graph showing the proportion of each type of motion (Figure 7c): in all cases restricted or confined motion amounted to more than 50% of all occurrences, the rest corresponding to simple Brownian motion or undetermined trajectories. No particle trajectories were observed to correspond to directed motions.

The MSD was also followed in whole cells (cf. Fig. 4) analyzing all particles with the software Localizer, thus including a very high number of trajectories. Table 2 lists the MSD for the different experimental conditions, i.e. between 18,000 and 42,000 particles for BTX-labeled samples and 15,000–100,000 particles for mAb-labeled samples.

**Table 2:** Mean square displacement (MSD) of AChR particles in samples labeled with Alexa^488^α-BTX or with anti-AChR monoclonal antibody (mAb210) followed by staining with Alexa^488^-labeled secondary antibody, with or without treatment with 15 mM methyl-β-cyclodextrin (CDx).

Experimentalcondition	Total No. of particles(in all frames)	No. of frames analyzed todetermine trajectory	Total No.compound tracks analyzed	Mean SquareDisplacement (µm^2^)
BTX Control	42,625	15	1702	0.0589±0.0016
		30	1530	0.0936±0.0028
		60	1462	0.1550±0.0056
BTX CDx	18,728	15	1572	0.0874±0.0079
		30	1120	0.1597±0.0177
		60	862	0.2255±0.0206
mAb Control	15,297	15	1476	0.0436±0.0032
		30	1126	0.0671±0.0049
		60	861	0.0974±0.0077
mAb CDx	104,227	15	956	0.0242±0.0019
		30	620	0.0388±0.0031
		60	388	0.0668±0.0105

Analyzed with the software Localizer [34].

### Distribution and degree of aggregation of AChR particles

**Figure 8:**
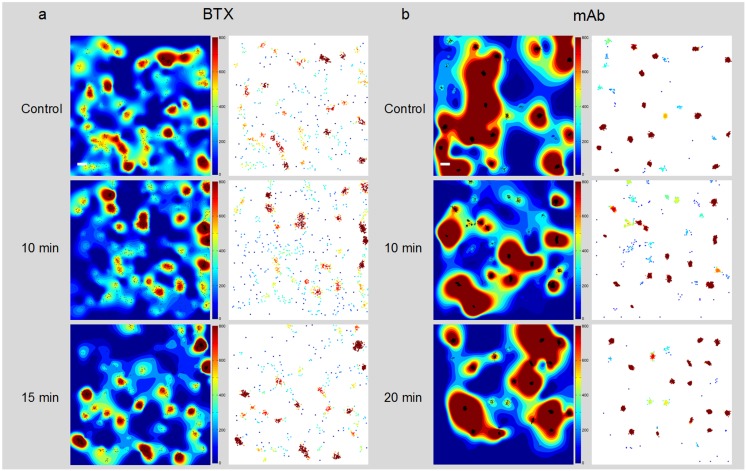
Time-dependent evolution of the quantitative cluster maps. a) Alexa488-α-BTX labeled AChR particles imaged with TIRF microscopy in CHO-K1/A5 cells. The left column shows the interpolated cluster maps resulting from local-point pattern analysis of 4×4 µm regions in control and CDx-treated cells at the indicated intervals (10 min, 15 min). The maps, based on Ripley’s K-function [39] provide a graphical representation of the degree of aggregation of particles (black dots) per unit area in the entire series of frames. The threshold radius for assigning cluster status to a group of particles is set at 200 nm. The right column corresponds to the map of clustered BTX-stained particles, pseudocolored according to relative brightness of the detected particles. b) Time-dependent evolution of the cluster maps of mAb-crosslinked AChR particles. The left column corresponds to the interpolated cluster map based on Ripley’s K-function applied to CHO-K1/A5 cells labeled with primary anti-AChR monoclonal antibody (mAb210) followed by staining with Alexa^488^-labeled secondary antibody. The right column shows the map of clustered AChR particles pseudocolored according to brightness. Scale bar: 0.2 µm.

This was quantitatively analyzed next using various approaches. First, we studied the spatial distribution of localized BTX- or mAb-labeled particles in manually selected areas of TIRF movies recorded from live CHO-K1/A5 cells (Figures 8a–b and Table 3). Ripley’s K-function [36], [37] analysis allows one to examine the spatial organization of the particles by comparing their bidimensional point distribution with patterns of complete spatial randomness. The analysis takes into account the distances between all points in the region of interest. Clusters were defined by a maximum nearest neighbor interparticle radial separation of 200 nm. This dimension is at the limit of the lateral resolution of the TIRF microscopy employed but is validated, i.e. physically meaningful- by the dimensions of the AChR nanoclusters resolved by STED microscopy [20]. Figure 8a shows the time-dependent evolution of the quantitative cluster maps of BTX-labeled AChR particles in control and 10 min and 15 min after CDx-treatment of the cells, respectively. The maps [39] graphically categorize areas of particle isodensity; discrete “hot spots” showing the highest degree of particle aggregation can be clearly identified in the two-dimensional projections stemming from the entire series of frames (Figure S11). The right column in Figure 8a depicts particles sorted according to their relative brightness. The clusters of highly bright particles identified by this criterion match the clustered regions sorted by positional recognition in the left column of Figure 8a. The number of clustered AChR particles at 10 min CDx treatment was significantly lower than that of the control, whereas at 15 min values similar to those of the control appear to be restored (Table 3).

**Table 3:** AChR particle and cluster statistics from the time-series experiments.

Experiment	Total number of particles	Particles in clusters	Brightness (a.u.)
BTX control	938.1±214^a^	895.8±209 (95.1%)^a^	727.7±86^a^* (n = 22)
BTX CDx (10 min)	514.4±192^b^*	470.6±182 (91.4%)^b^*	1075.7±196^b^ (n = 17)
BTX CDx (15 min)	931.2±262^c^	886.6±269 (95.2%)^c^	1194.6±163^c^ (n = 16)
mAb control	736.7±474^d^*	680.7±455 (92.3%)^d^*	1730.6±500^d^ (n = 20)
mAb CDx (10 min)	8930.8±3200^e^*	8859.9±3183 (99.2%)^e^*	6614.6±1273^e^* (n = 17)
mAb CDx (20 min)	5521.3±2776^f^	5487.3±2761 (99.34%)^f^	2699.5±455^f^ (n = 15)
mAb CDx (40 min)	1151.1±995^g^	1134.3±990 (98.54%)^g^	3023.5±302^g^ (n = 18)

The threshold radius for assigning cluster status to a group of particles was set at 200 nm.

Symbols denote statistically significant differences (Kruskal Wallis test, P<0.05).

Total number of particles:

b exhibited statistically significant difference with a and c.

d exhibited statistically significant difference with e, f and g.

Particles in clusters:

b exhibited statistically significant difference with a and c.

d exhibited statistically significant difference with e, f and g.

Brightness:

a exhibited statistically significant difference with b and c.

e exhibited statistically significant difference with d, f and g.

In the case of mAb-crosslinked AChR particles, the most striking observation was the much larger size of the clusters (in comparison to BTX-labeled samples), many of which extended in linear patters (Figure 8b). The quantitative cluster maps also showed a time-dependent change at 10 min, albeit quantitatively very different (Figure 8b) from that of BTX-labeled particles. As shown in Table 3, the number of particles in clusters increased by more than a factor of 10 upon 10 min CDx treatment of mAb-crosslinked receptors. The density of particles remained high at subsequent intervals explored, but the “burst” of clustering apparently takes place at 10 min. In addition, the graphical cluster maps identified much larger isodensity areas in mAb-crosslinked samples than those observed in BTX-labeled samples (Figure 8b).

**Figure 9:**
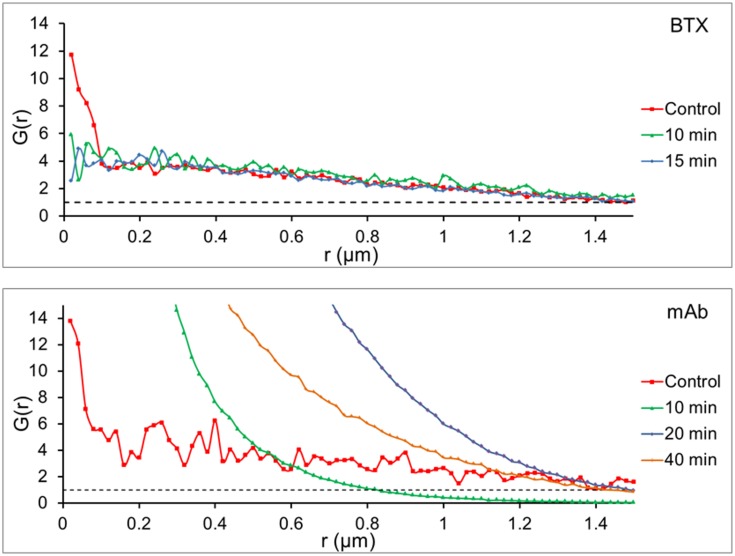
Pair correlation function analysis. a) CHO-K1/A5 cells stained with Alexa^488^-α-BTX under control conditions and at 10 or 15 min after acute application of 15 mM CDx as in Figure S1a. (b) CHO-K1/A5 cells stained with anti-AChR monoclonal antibody (mAb210) followed by staining with Alexa^488^-labeled secondary antibody, under control conditions and 10, 20 or 40 min after acute in situ application of 15 mM CDx as in Figure S1b. The pair correlation function *G(r)* identifies the length scale of the clustering [42], [47], [48]. *G(r)* was corrected for density effects.

Another analytical tool for assessing particle clustering is the so-called pair correlation function, *G(r),* as shown in Figure 9 and Table 3. The pair correlation function *G(r)* estimates the probability of finding another particle at a distance *r*, and compares the data to the expected values for a random distribution of particles. Values of *G(r)* that are >1 indicate non-random distribution, which can be assumed to correspond to particle clustering [42], [43], [47], [48]. The *G(r)* plot (Figure 9a), corrected for density effects, corroborates the non-random distribution of BTX-labeled AChR particles under control and cholesterol-depleted conditions and provides additional evidence of the differences between monoliganded (BTX) and crosslinked (antibody-labeled) samples. The pair correlation function was also found to change as a function of time: for cells labeled with BTX (Figure 9a) the detected particles exhibited a high degree of clustering at very short length scales in the control sample as compared to particles in cells treated with CDx. The *G(r)* function extended its non-random, clustered pattern up to a radius >1 µm.

In the case of mAb-stained cells, a marked difference was observed between control and cholesterol-depleted samples. The values of *G(r)* fluctuated between 3 and 6, i.e. they exhibited a non-random spatial distribution, for radii up to 1.4 µm (Figure 9b), whereas the *G(r)* curves for 10, 40 and 20 min CDx treatment indicated very strong inter-particle correlations at decreasing length scales (Figure 9b). The *G(r)* corresponding to 10 min CDx treatment sharply declined exponentially, its behavior becoming random for radii larger than 0.8 µm. The 40 min and 20 min CDx samples exhibited a laxer decline, and in both cases particles remained non-randomly distributed for radii below 1.4 µm, with a robust correlation for distances below 1 µm. The strongest correlation of the *G(r)* function was observed at 20 min CDx treatment.

## Discussion

AChRs are ligand-activated cation channels that mediate fast excitatory neurotransmission in the peripheral and central nervous systems. The extent to which lipid platforms modulate the supramolecular organization of the AChR is still not fully resolved.

In the present work, the 2-D mobility of the membrane-bound AChR at high densities and its dependence on membrane cholesterol levels and clustering mediated by antibody crosslinking were studied using a combination of fluorescence microscopy and single-particle analytical techniques. Firstly, fluorescence TIRF microscopy was chosen to select only cell-surface AChR molecules, thus exploiting the inherent advantages of the evanescent wave excitation, in particular its enhanced axial resolution (see e.g. [49]). The non/propagating (evanescent) field created by TIR illumination excites fluorophores only at the interface between the microscope coverslip and the aqueous medium, providing a roughly 4-fold improvement in signal-to-noise ratio because of the significant drop in excitation occurring within a shallow volume beyond the evanescent wave inside the cell. This feature makes TIRF ideal for studying fluorescent-labeled receptors at the basal membrane of living cells growing on a glass substrate. We have previously characterized the endocytic mechanism of cell-surface AChRs in CHO-K1/A5 cells [50], operating in the time scale of hours and with a negligible degree of recycling within the initial 2 h. The slow internalization kinetics and the lack of recycling thus provide a useful time window of more than one hour, thus ensuring that one is recording the motional activity of membrane-bound receptors without the “spurious” contribution of newly incorporated receptor molecules or the disappearance of receptors by endocytic internalization.

One relevant aspect of the present work is that the mobility of AChR clusters could be followed at high particle densities, a situation which tends to mimic conditions met at developing synapses, not the fully developed neuromuscular junction. In recent work on α7 AChR in cultured hippocampal neurons, SPT analysis required labeling of only a small fraction of receptors with quantum dot-coupled α-BTX [51]. Furthermore, we have employed streamed image acquisition of cell membrane-bound AChR nanoclusters, limited only by the speed of the CCD camera. The 2-D regions were analyzed in terms of trajectories of individual nanoclusters. The kinetics of translational mobility, speed, path lengths, etc., the lateral diffusion coefficient, *D*, the relative proportion of mobile and immobile fractions, and the trajectories themselves were analyzed under different experimental conditions. Analysis of the particle trajectories at high particle densities was made possible using the software U-track developed by Jaqaman et al. [26], an open code supporting the integration of personalized algorithms and able to handle relatively large amounts of data efficiently and within acceptably short times with standard personal computer power (see Material and Methods). U-track is a multiple-particle tracking Matlab software designed to follow trajectories in fields densely populated by particles, a condition often found with cell-surface receptors expressed at high densities in mammalian cells. Furthermore, U-track closes the gaps in particle trajectories resulting from detection failure, and captures particle merging and splitting events resulting from occlusion or genuine aggregation and dissociation events.

### Antibody-mediated AChR crosslinking dramatically restricts receptor mobility

In agreement with AChR crosslinking studies in rat myotubes in primary culture [52] and recent work from our laboratory employing FRAP techniques in CHO-K1-A5 cells [53] antibody-induced crosslinking resulted in a drastic diminution of AChR mobility. Instead of the long particle walks observed with a monovalent ligand such as α-BTX (Figures 1–2 and 4), the motion of antibody-crosslinked AChR particles was restricted to much shorter trajectories confined within relatively small areas (Figures 2, 6 and 7). In a recent work, Sieber et al. [54] determined that plasma membrane clusters of syntaxin depend on weak homophilic protein-protein interactions, and that syntaxin molecules in these clusters are dynamically exchanged with freely diffusing molecules.

### Effect of cholesterol on AChR nanocluster mobility

We also analyzed the behavior of the AChR upon manipulation of membrane cholesterol. In previous work we determined that cholesterol depletion of CHO-K1/A5 cells had multiple functional consequences: i) lowering cholesterol affected AChR channel properties, producing gain-of-function, as measured by mean open time distribution in single-channel patch-clamp recordings, whereas cholesterol enrichment had the opposite effect [19]; ii) using FRAP, we found that CDx treatment reduced the fraction of mobile AChRs from 55 to 20% [53]. Concomitantly, the fluorescence recovery of the toxin-labeled receptor observed in FRAP experiments was clearly slower (2.1±0.7×10^−11^ cm^2^ s^−1^) than in control cells (4.4±0.4×10^−11^ cm^2^ s^−1^), and cholesterol enrichment had the opposite effect [53]; iii) when the plasma membrane of CHO-K1/A5 cells was depleted of cholesterol the rate of endocytosis was dramatically enhanced [19]; iv) the endocytic pathway changed under low cholesterol conditions [55].

A series of recent publications emphasizes the importance of membrane cholesterol in the biogenesis and stability of AChR clusters in vivo and in vitro. Cholesterol was found to influence the formation of micron-sized AChR clusters induced by agrin [13]. Signaling via the agrin/MuSK complex and interaction between the receptor and rapsyn appears to involve lipid platforms [17]. Using Laurdan two-photon fluorescence microscopy Stetzkowski-Marden et al. [15] concluded that AChR clusters reside in ordered membrane domains, a biophysical property characteristic of solid-like lipid domains. Willmann et al. [16] proposed that cholesterol-rich lipid microdomains and Src-family kinases both contribute to stabilizing the postsynaptic apparatus. In our experimental clonal cell line, CHO-K1/A5, there are no AChR-clustering proteins such as rapsyn and tyrosine kinases, and therefore homophilic protein-protein interaction and links with the actin cytoskeleton are more likely candidates for maintaining the AChR nanocluster assemblies.

Several FRAP studies have shown that cholesterol depletion affects the mobility of various proteins at the plasma membrane although the nature, extent and sign of the changes remain a contentious subject. In FRAP experiments performed on cells treated with Mevinolin, a statin that inhibits cholesterol biosynthesis, we found that AChR mobility was affected in a manner similar to that reported here using CDx-mediated acute cholesterol depletion [53]. On the basis of these observations, we can conclude that plasma membrane fluidity is not the main factor determining AChR mobility. Some authors reported that the mobility of raft- and non-raft resident proteins decreases when cholesterol is removed from the plasma membrane [56], [57]. Restricted diffusion of membrane proteins upon cholesterol depletion is believed to result from the formation of solid-like clusters in the membrane [58], [59]. Using single-molecule tracking methods, Orr et al. [60] found that cholesterol depletion produces confinement of the epidermal growth factor receptor and human epidermal growth factor receptor 2 mobility, whereas cholesterol enrichment extended the boundaries of the mobility-restricted areas. In contrast, other authors observed an increase in the lateral mobility of the raft-resident proteins CD44 and wild-type GFP-H-Ras after cholesterol depletion [61], [62]. Removal of cholesterol, particularly with CDx, not only alters membrane viscosity but can also hinder membrane protein diffusion [63].

### 2-Dimensional distribution and clustering of AChR particles

Quantitative local point-pattern analysis based on Ripley’s K-function was applied to calculate maps of particle cluster­ing (Figure 8 and Table 3). The thresholded color-coded cluster maps not only constituted a straightforward visual inspection tool, but provided relevant cluster statistics [39], [40], [64]. Following Ripley’s criteria, by comparing the point distribution of particles with patterns of complete spatial randomness, the experimentally identified particles were found not to be randomly distributed but organized in clusters. This type of arrangement changed as a function of time of exposure to CDx, reaching a maximum at 10 min treatment both for BTX- and mAb-labeled samples (Figure 8 and Table 3), in accordance with the time-dependence observed in SPT data (Table 1). The density and size information extracted from pair correlation analysis is an average over many clusters. Pair correlation analysis identifies individual clusters based on a maximum distance, and therefore can be used to measure the distribution of individual clusters [65]. Recent studies on AChR SPT in myoblasts show that the majority of the receptors were immobile, with 20% of the receptors exhibiting restricted diffusion in small domains of about 50 nm. In myoblasts devoid of rapsyn, the fraction of mobile AChRs increased considerably, accompanied by a 3-fold decrease in the immobile population of in comparison to rapsyn-expressing cells. Half of the mobile receptors were confined to domains of about 120 nm [66].

### Functional bearing of AChR mobility and its modulation by cholesterol and actin meshwork

Recently, Berg and coworkers [24] reported that CDx treatment increased the mobility of neuronal-type α7 AChRs but not that of α3 AChRs in central nervous system synapses, concluding from these and other data that AChR mobility is receptor-subtype specific. They also observed that CDx treatment rendered half of the immobile α3 AChRs mobile without changing the proportion of the immobile α7 AChRs. They further reported that disruption of PDZ-containing scaffolds or of actin filaments in chick ciliary ganglion neurons increased the mobility of α7 AChRs [24], as expected from the wealth of evidence on the role of the actin and PDZ-scaffolds in maintaining synapse, and in particular dendritic spine, architecture [67]. AChR mobility displays a strong dependence on cytoskeletal integrity [68]–[70] in myotubes and in the neuromuscular junction. Using FRAP and FCS, two ensemble methods suitable for interrogating membrane protein mobility, we have recently reported that the mobility of the adult murine muscle-type AChR heterologously expressed in the clonal cell line CHO-K1/A5 developed in our laboratory [25], [71] is also dependent on cytoskeletal integrity [53]. We have shown that in these cells the AChR aggregates in the form of very small, nanometer-sized clusters [20], although the cells lack rapsyn and other scaffolding or receptor-anchoring proteins. This allows one to study the inherent mobility/dynamics of AChR assemblies in the absence of receptor-anchoring molecules and provides a convenient minimalist approach to define the crosstalk between the AChR protein and the neutral lipid, cholesterol. Moreover, the results reported here reinforce the conclusion of Fernandes et al. [24] on the receptor-subtype specificity of the motional regime adopted by different AChRs. The muscle-type AChR studied here is inherently mobile; a modest proportion (20%) attains immobility by antibody crosslinking. More importantly, a dramatic (83%) but transient increase in the percentage of immobile receptors was observed upon cholesterol depletion of the cells, especially during the initial 10 min. The percentage of stationary particles fell thereafter to 57% (20 min) and 27% (40 min) when cells having antibody-crosslinked receptors were treated with CDx. Thus, antibody crosslinking and cholesterol depletion exhibited a mutually synergistic, time-dependent effect.

## Conclusions

The present work shows that the mobility of the adult muscle-type AChR at the cell surface is modulated by the size of its supramolecular organization–the nanocluster [20], and hence by the number of receptor units in the assembly. It has been previously reported that in one cell, a single species of protein can have one subset undergoing Brownian diffusion whereas other subsets undergo confined or anomalous diffusion [72]. Secondly, the neutral lipid cholesterol is also shown to modulate cell-surface diffusion of AChR nanoclusters. The density of AChRs at the synapse is a consequence of the dynamic equilibrium between synthesis, internalization, recycling and diffusion in/out of the synaptic area [73], [74]. Cholesterol, synergistically coupled to the size of the AChR nanoclusters, could thus exert homeostatic control over the maintenance of receptor levels and the dynamics of the AChR supramolecular assemblies at the cholinergic synapse.

## Supporting Information

Figure S1 Fluorescent particles in control and cholesterol-depleted CHO-K1/A5 cells. a) Control CHO-K1/A5 cells stably expressing adult muscle-type AChR were labeled with AlexaFluor488-α-BTX, excited with a 488 nm Ar laser, and imaged with TIRF microscopy at a sample rate of 7.5 Hz (133.25 ms/frame). The image corresponds to the 8.8 s time frame. Bar: 10 µm. b) CHO-K1/A5 cells treated with 10 mM CDx for 20 min. The image corresponds to the initial frame (t = O) of a time-series acquired at a sample rate of 7.9 Hz (127.23 ms/frame). c) Kinematic representation of particles corresponding to the control CHO-K1/A5 cell in (a). Small arrows point to the beginning and termination of traces corresponding to very short events.
(TIF)

Figure S2An example of simple Brownian diffusion. Three basic conditions were simulated: simple Brownian, directed and confined motions. The function *rand*() in Matlab was used to generate the initial conditions on the basis of random numbers generated with a uniform standard distribution within the interval (0,1). The value of the mean path (<P>) of the particle in the simple Brownian motion and the value of the dispersion of P (ΔPmax) is taken into account up to the n-th frame in the form: 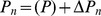; such that 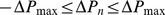 The particular case of Brownian stationary motion only differs in the value of <P>; the particle trajectory is the same. The n-th step of the trajectory (n-th temporal sample) follows the form: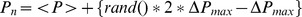, 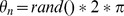, 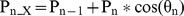, 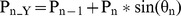
_._
(TIF)

Figure S3 Displacement vector and the bisector of the aperture angle θmax used to model directed diffusion. In order to model directed diffusion a displacement vector (DP) was added. Its module is given by *rand*()*dPmax, where dPmax is the maximal displacement in a frame, i.e. the one determining vmax = dPmax/δt, within the environment of the direction θ0.
(TIF)

Figure S4Directed diffusion. In directed diffusion the displacement is made up of a Brownian component as in the simple Brownian case, plus a drift component, dP, with a module given by: 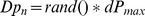, If one takes: 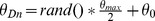, the components for the n-frame will thus be given by, (see figure S4): 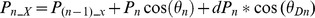. 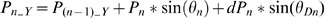.(TIF)

Figure S5 An example of directed diffusion.
(TIF)

Figure S6 An example of a trajectory in the confined motion mode. The expressions are similar to those of the simple Brownian case, except that the particle moves in a Brownian fashion within a circumference of radius R.
(TIF)

Figure S7 Combined motional regimes. A complex single trajectory displaying the three main motional regimes: simple Brownian (red), confined Brownian (green), and directed (blue).
(TIF)

Figure S8 Simulation of the MSD as a function of *Δt*. a) The simulation comprised a total of 300 individual trajectories, of which 100 exhibited a simple Brownian motion, 100 directed Brownian motion, and the remaining 100 particles restricted (confined) Brownian diffusion. Figure S8b shows in greater detail the differences between the three types of motion: the linear tendency corresponding to particle diffusion following simple Brownian motion, the rapidly growing curves with quadratic behavior which follow a defined direction (directed Brownian motion), and the remaining curves corresponding to particles diffusing within a confined region, as revealed by their associated essentially constant MSD for high *Δt* values.
(TIF)

Figure S9 Example of an experimental MSD. The linear (red) and quadratic (blue) fits to the MSD data points are shown, together with their corresponding residuals (lower panel).
(TIF)

Figure S10 Automatic MSD analysis. Mean-square displacements were also analyzed automatically using the software Localizer. See Table 1 for details.
(TIF)

Figure S11 Cluster analysis of AChR particles. Graphical cluster analysis based on Ripley’s K-function [39] provided a straightforward visualization of the aggregation of AChR particles at the cell membrane. Local-point pattern analysis [40] rendered additional information on the incidence of “hot-spots” with the highest density of particles and their 2-dimensional localization. This figure shows the sequence from raw TIRF images to the graphical rendering of cluster distribution. a) TIRF image of CHO-K1/A5 cells stained with Alexa488-α-BTX. The first frame of a movie comprising 1024 frames is shown. b) The output of the QuickPALM reconstruction procedure [33] rendered the totality of particles thresholded above a certain brightness level in the entire movie. The area outlined in red corresponds to a 7.5×7.5 µm region manually selected for further analysis. (c) Cluster map resulting from local-point pattern analysis [40] of the area outlined in red in (b). Visual identification of “hot spots” of clustered particles (black dots) in the entire series of frames. (d) Graphical cluster map based on Ripley’s K-function [39], pseudocolored according to relative fluorescence intensity in each individually detected particle.
(TIF)
