# Supplementary figures and images for: Transient Cholesterol Effects on Nicotinic Acetylcholine Receptor Cell-Surface Mobility
Source: PLoS One. 2014 Jun 27;9(6):e100346. doi: 10.1371/journal.pone.0100346 (PMC4074099; doi:10.1371/journal.pone.0100346)

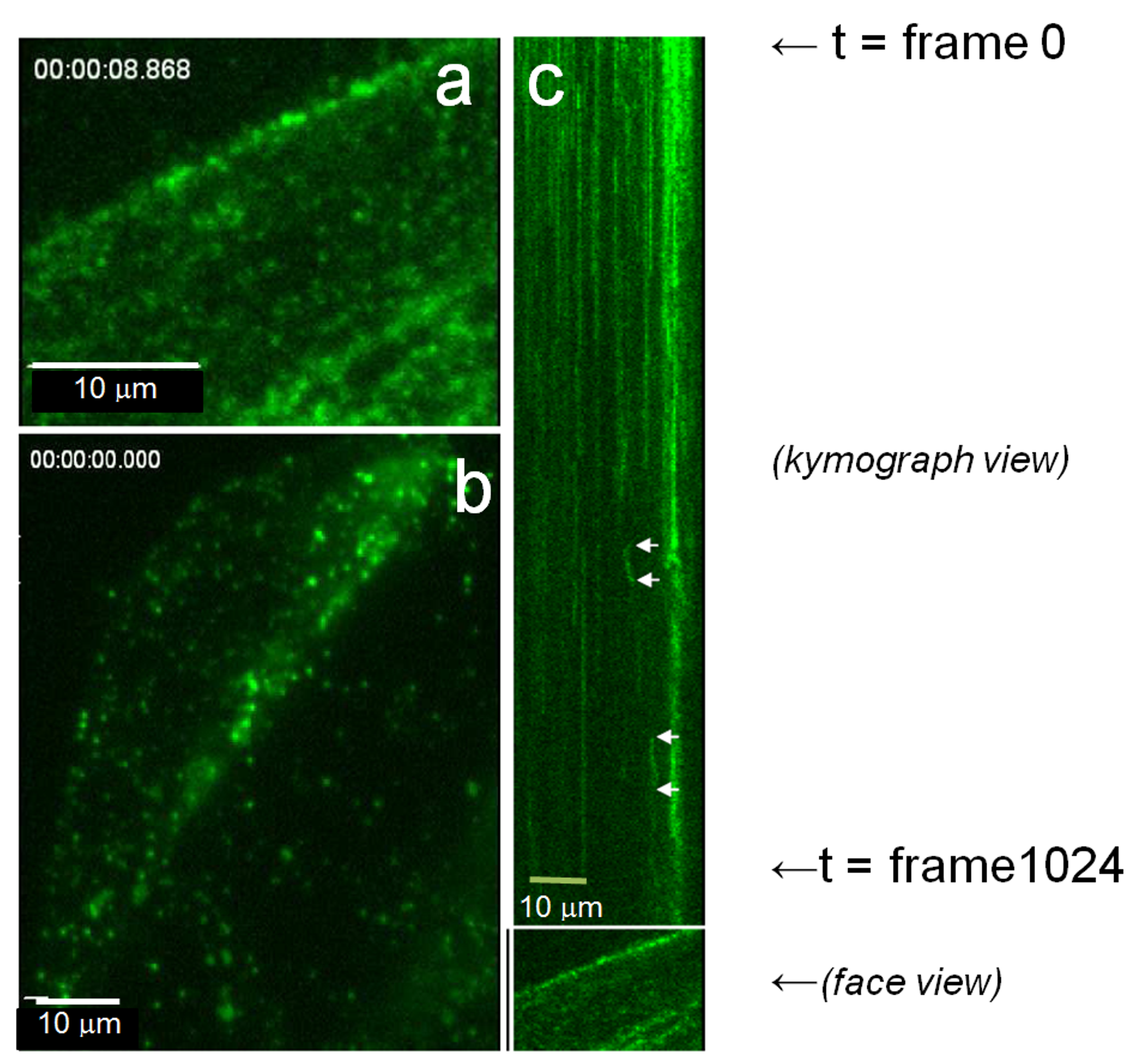

Supplement: Figure S1 — Fluorescent particles in control and cholesterol-depleted CHO-K1/A5 cells. a) Control CHO-K1/A5 cells stably expressing adult muscle-type AChR were labeled with AlexaFluor488-α-BTX, excited with a 488 nm Ar laser, and imaged with TIRF microscopy at a sample rate of 7.5 Hz (133.25 ms/frame). The image corresponds to the 8.8 s time frame. Bar: 10 µm. b) CHO-K1/A5 cells treated with 10 mM CDx for 20 min. The image corresponds to the initial frame (t = O) of a time-series acquired at a sample rate of 7.9 Hz (127.23 ms/frame). c) Kinematic representation of particles corresponding to the control CHO-K1/A5 cell in (a). Small arrows point to the beginning and termination of traces corresponding to very short events. (TIF) [file pone.0100346.s001.tif]

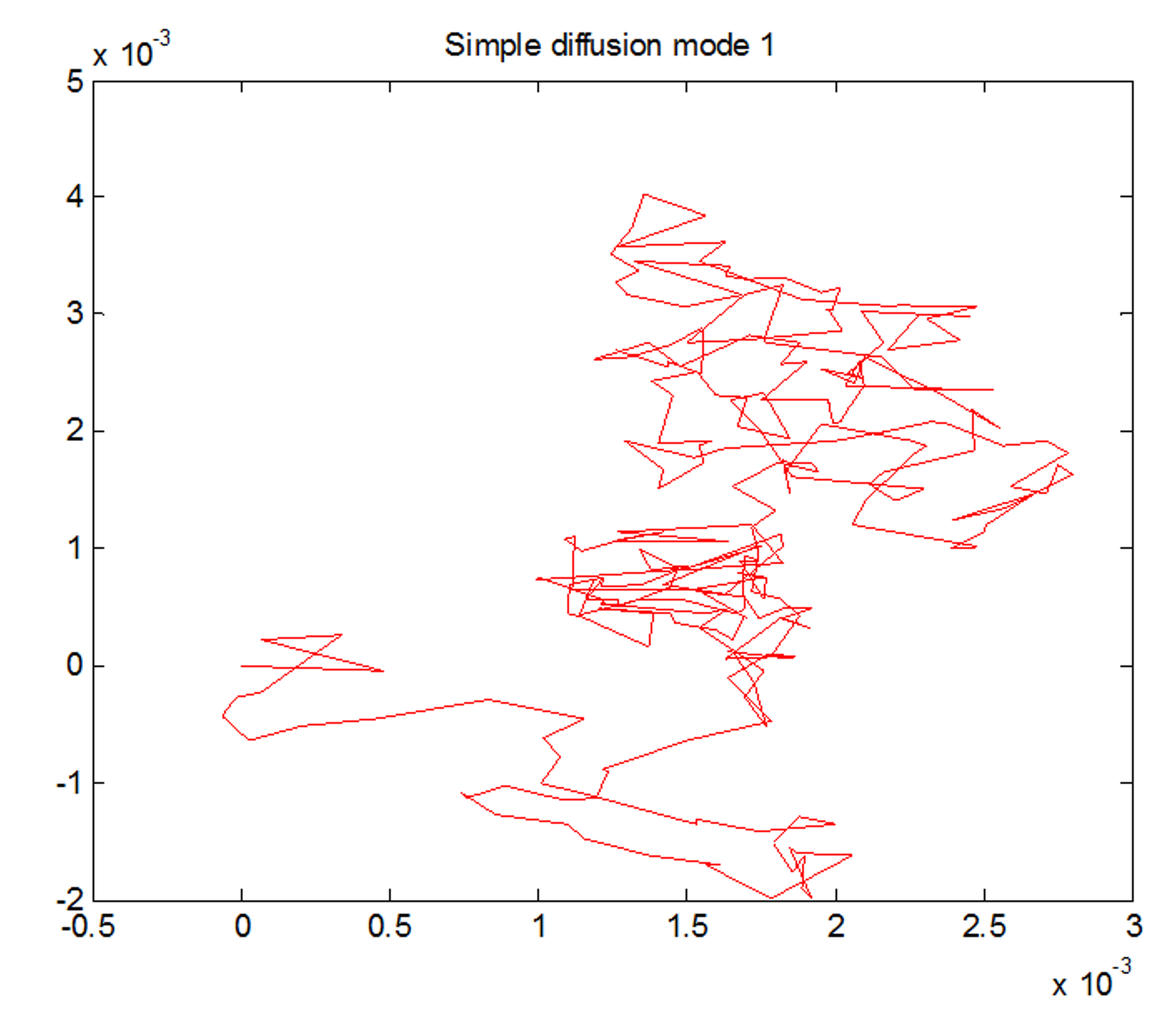

Supplement: Figure S2 — An example of simple Brownian diffusion. Three basic conditions were simulated: simple Brownian, directed and confined motions. The function rand() in Matlab was used to generate the initial conditions on the basis of random numbers generated with a uniform standard distribution within the interval (0,1). The value of the mean path () of the particle in the simple Brownian motion and the value of the dispersion of P (ΔPmax) is taken into account up to the n-th frame in the form: ; such that The particular case of Brownian stationary motion only differs in the value of ; the particle trajectory is the same. The n-th step of the trajectory (n-th temporal sample) follows the form:, , , . (TIF) [file pone.0100346.s002.tif]

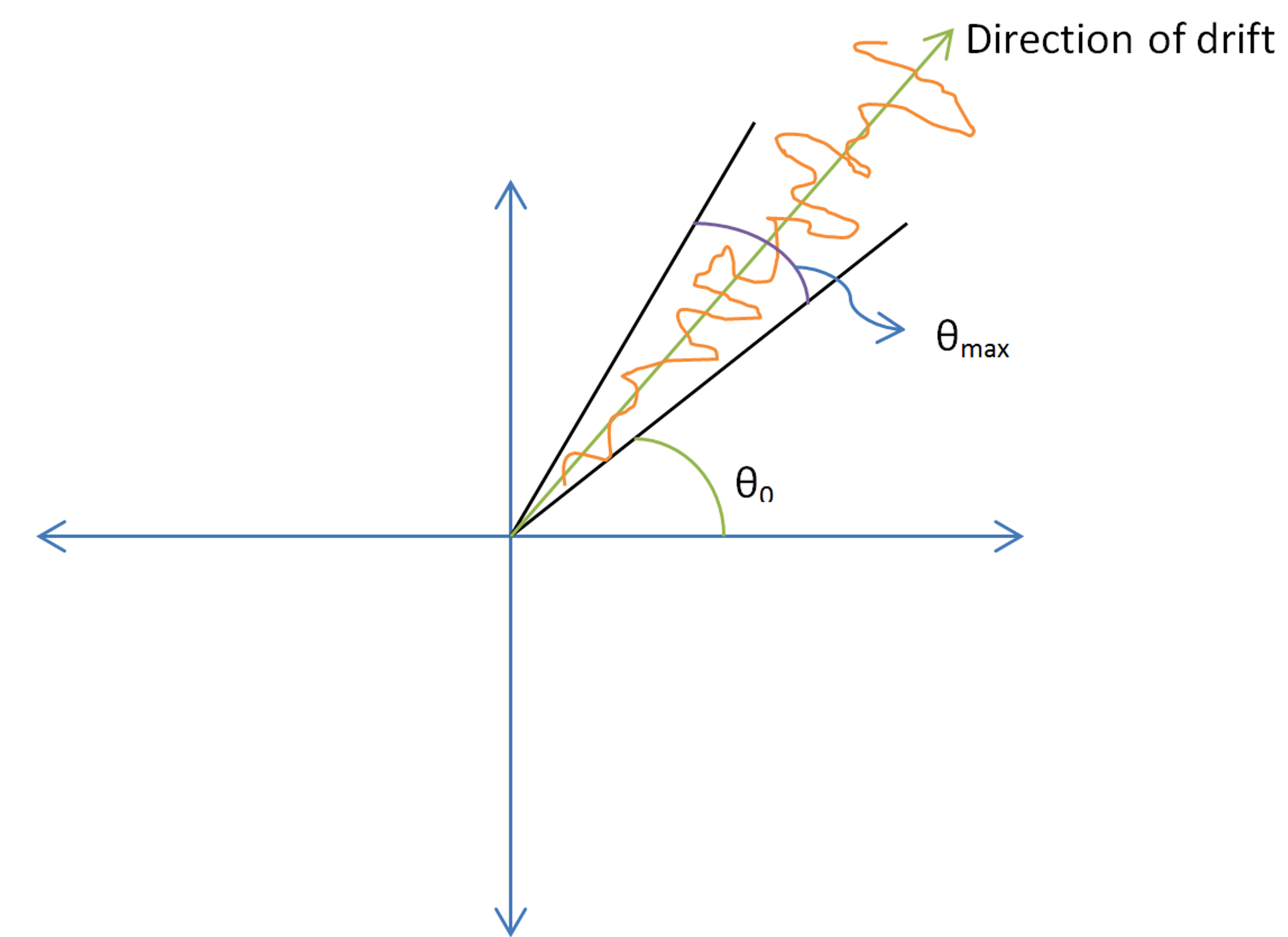

Supplement: Figure S3 — Displacement vector and the bisector of the aperture angle θmax used to model directed diffusion. In order to model directed diffusion a displacement vector (DP) was added. Its module is given by rand()*dPmax, where dPmax is the maximal displacement in a frame, i.e. the one determining vmax = dPmax/δt, within the environment of the direction θ0. (TIF) [file pone.0100346.s003.tif]

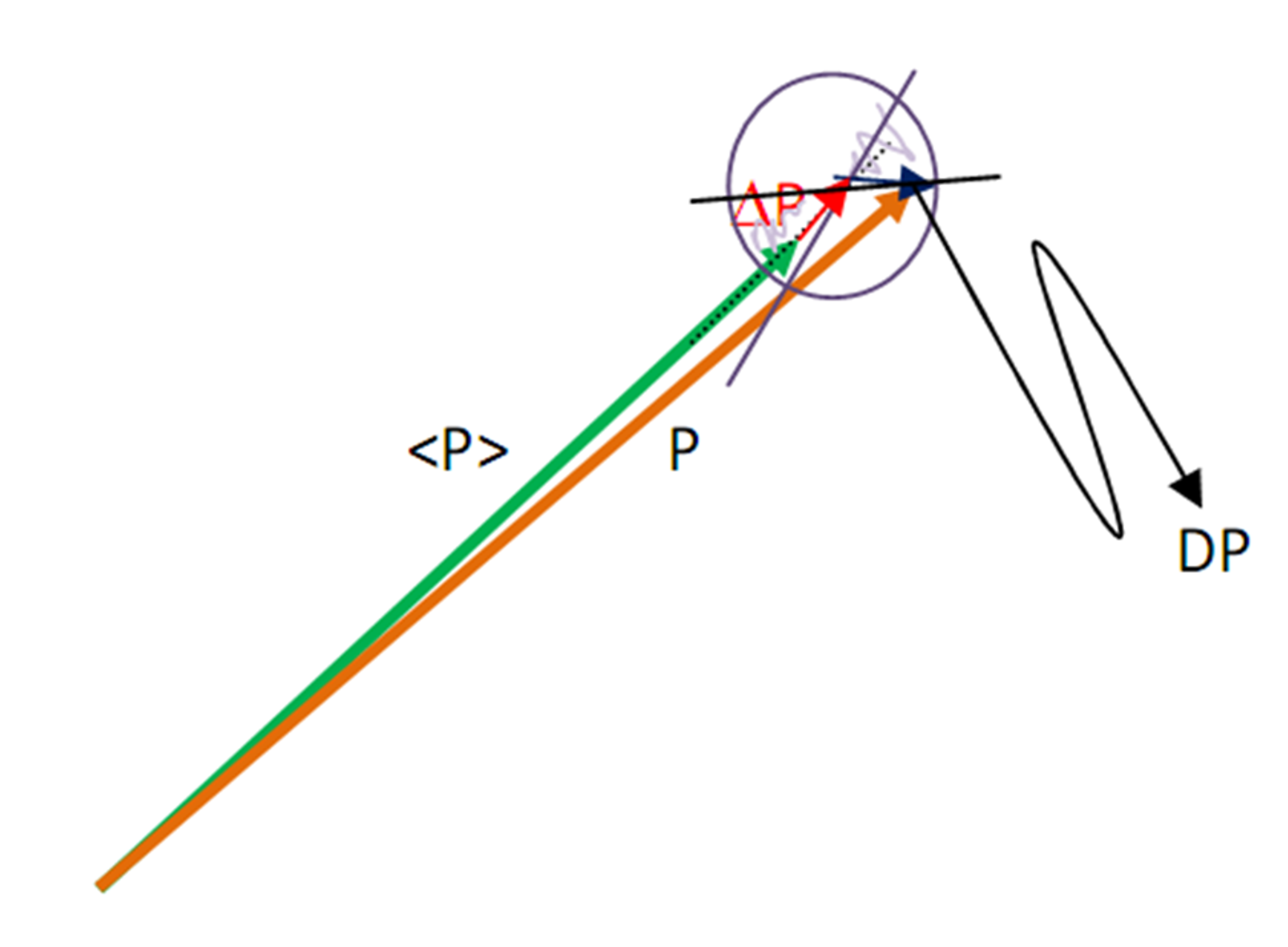

Supplement: Figure S4 — Directed diffusion. In directed diffusion the displacement is made up of a Brownian component as in the simple Brownian case, plus a drift component, dP, with a module given by: , If one takes: , the components for the n-frame will thus be given by, (see figure S4): . . (TIF) [file pone.0100346.s004.tif]

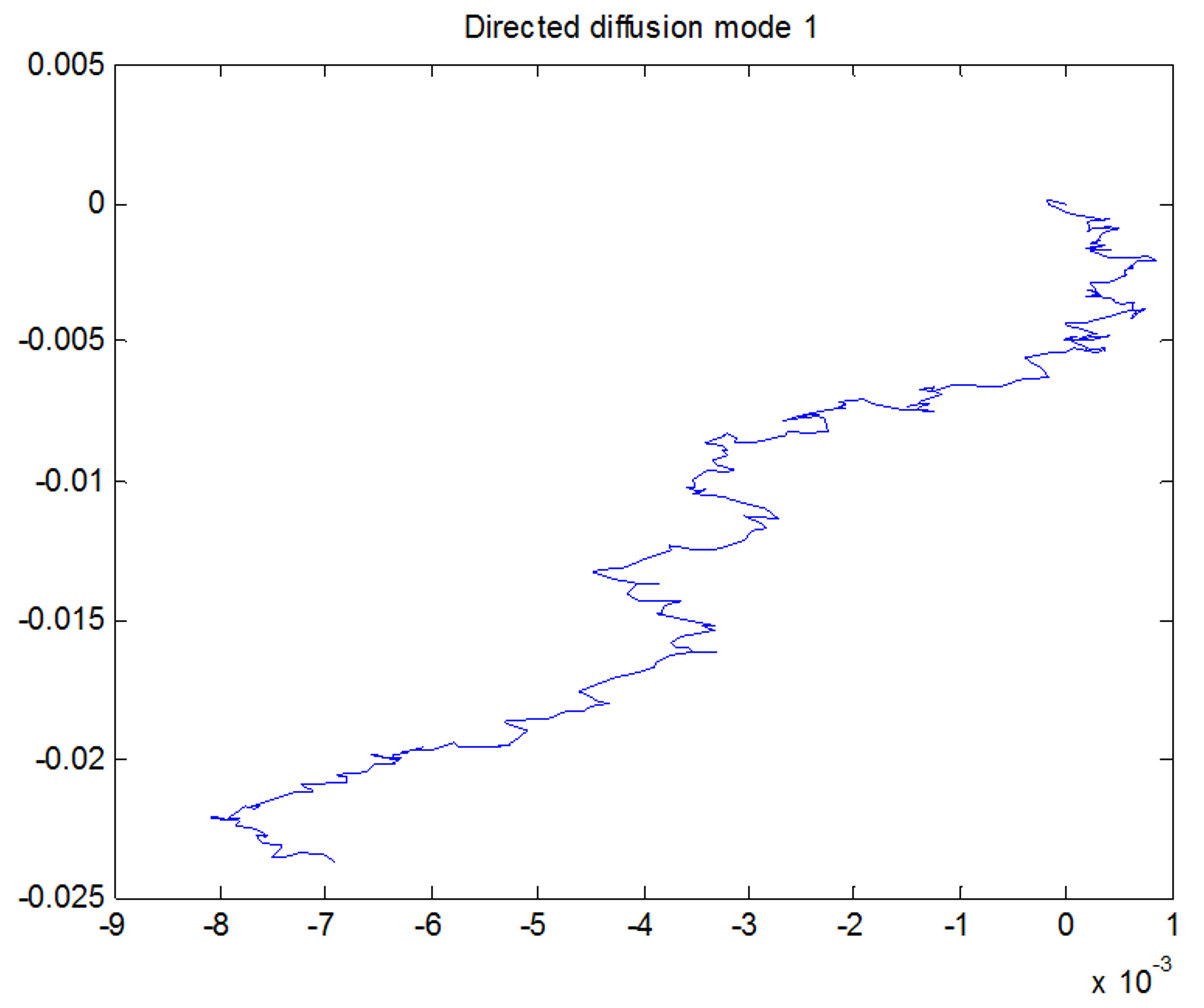

Supplement: Figure S5 — An example of directed diffusion. (TIF) [file pone.0100346.s005.tif]

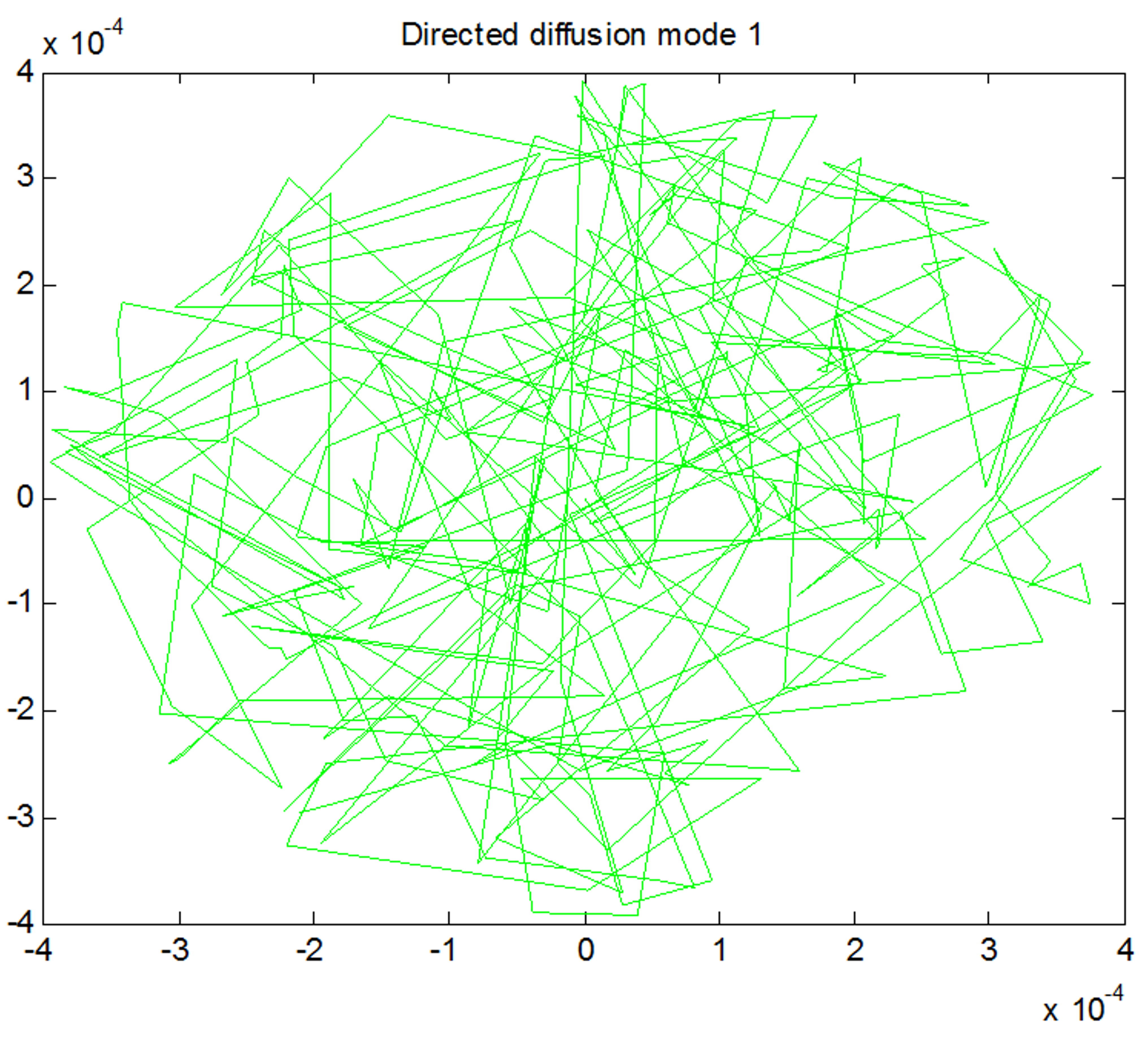

Supplement: Figure S6 — An example of a trajectory in the confined motion mode. The expressions are similar to those of the simple Brownian case, except that the particle moves in a Brownian fashion within a circumference of radius R. (TIF) [file pone.0100346.s006.tif]

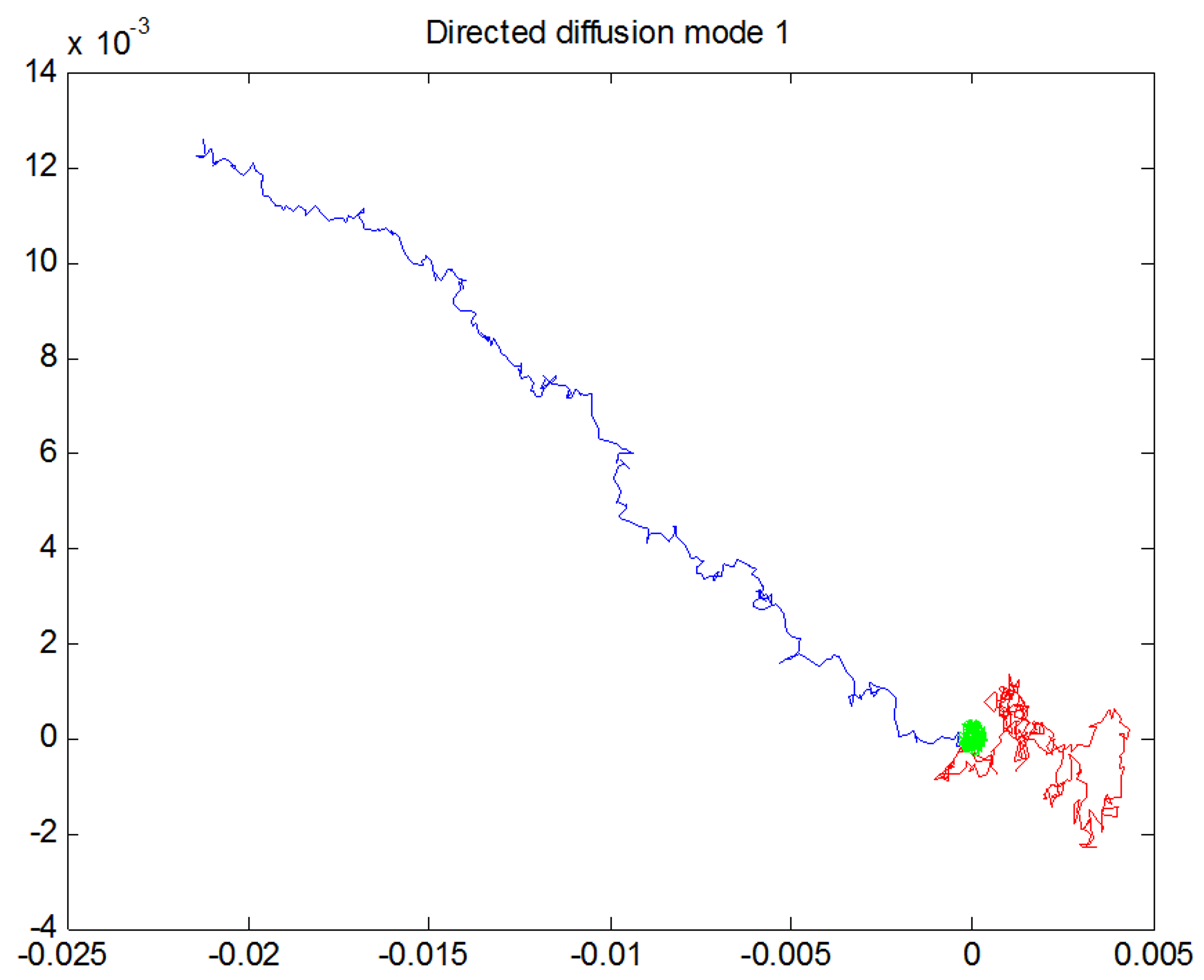

Supplement: Figure S7 — Combined motional regimes. A complex single trajectory displaying the three main motional regimes: simple Brownian (red), confined Brownian (green), and directed (blue). (TIF) [file pone.0100346.s007.tif]

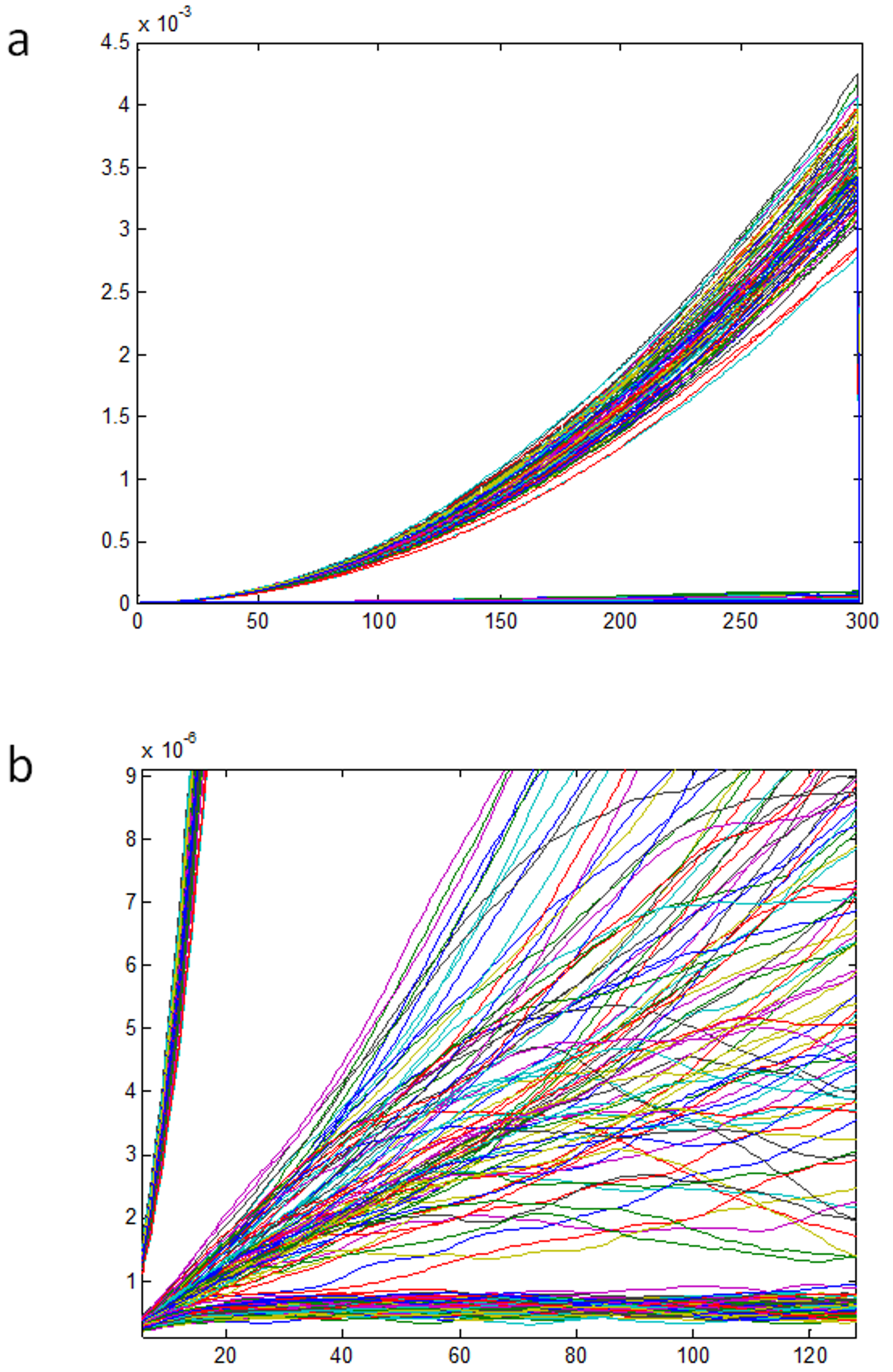

Supplement: Figure S8 — Simulation of the MSD as a function of Δt. a) The simulation comprised a total of 300 individual trajectories, of which 100 exhibited a simple Brownian motion, 100 directed Brownian motion, and the remaining 100 particles restricted (confined) Brownian diffusion. Figure S8b shows in greater detail the differences between the three types of motion: the linear tendency corresponding to particle diffusion following simple Brownian motion, the rapidly growing curves with quadratic behavior which follow a defined direction (directed Brownian motion), and the remaining curves corresponding to particles diffusing within a confined region, as revealed by their associated essentially constant MSD for high Δt values. (TIF) [file pone.0100346.s008.tif]

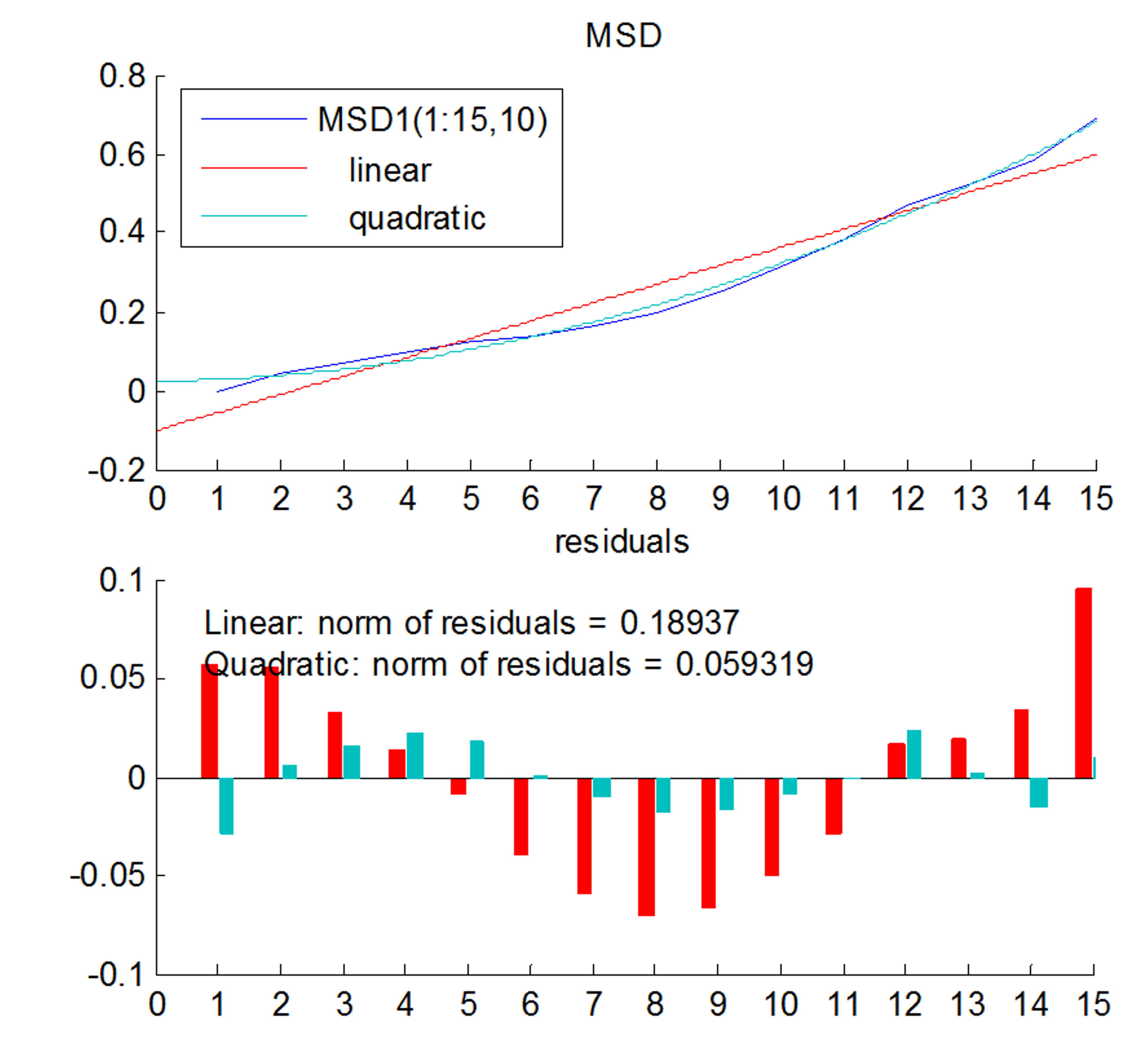

Supplement: Figure S9 — Example of an experimental MSD. The linear (red) and quadratic (blue) fits to the MSD data points are shown, together with their corresponding residuals (lower panel). (TIF) [file pone.0100346.s009.tif]

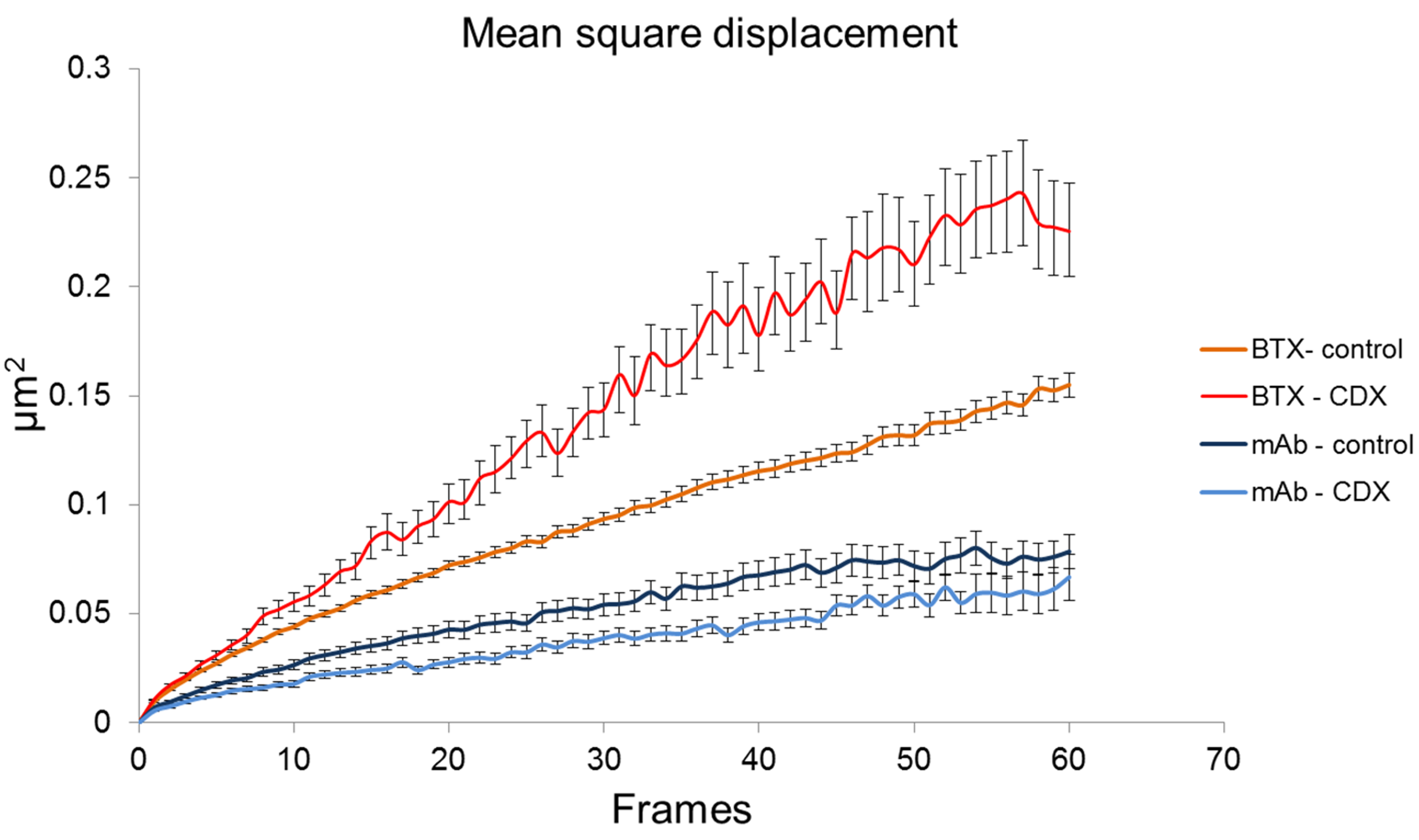

Supplement: Figure S10 — Automatic MSD analysis. Mean-square displacements were also analyzed automatically using the software Localizer. See Table 1 for details. (TIF) [file pone.0100346.s010.tif]

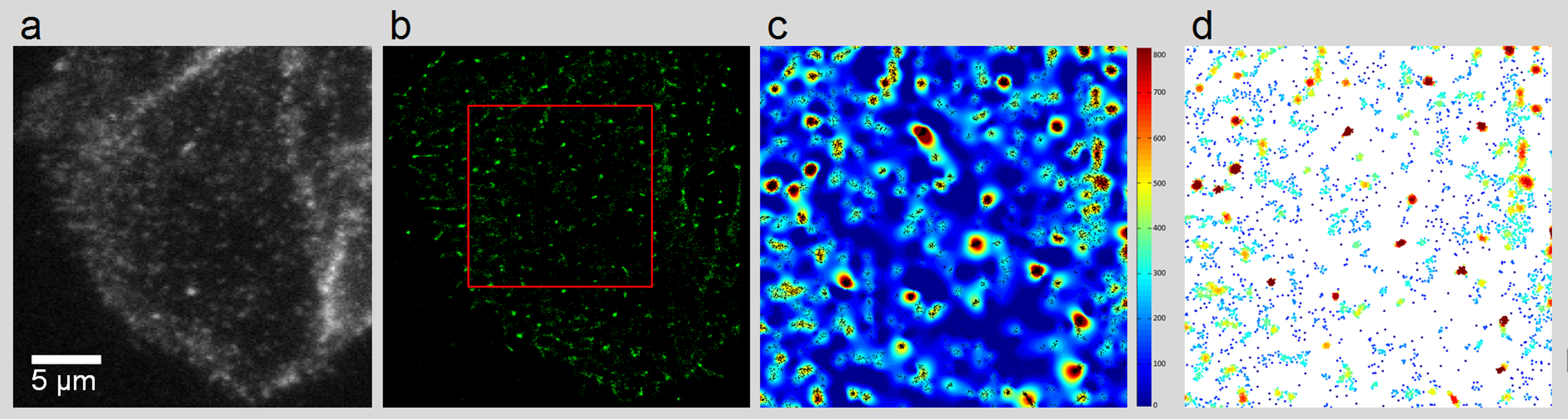

Supplement: Figure S11 — Cluster analysis of AChR particles. Graphical cluster analysis based on Ripley’s K-function [39] provided a straightforward visualization of the aggregation of AChR particles at the cell membrane. Local-point pattern analysis [40] rendered additional information on the incidence of “hot-spots” with the highest density of particles and their 2-dimensional localization. This figure shows the sequence from raw TIRF images to the graphical rendering of cluster distribution. a) TIRF image of CHO-K1/A5 cells stained with Alexa488-α-BTX. The first frame of a movie comprising 1024 frames is shown. b) The output of the QuickPALM reconstruction procedure [33] rendered the totality of particles thresholded above a certain brightness level in the entire movie. The area outlined in red corresponds to a 7.5×7.5 µm region manually selected for further analysis. (c) Cluster map resulting from local-point pattern analysis [40] of the area outlined in red in (b). Visual identification of “hot spots” of clustered particles (black dots) in the entire series of frames. (d) Graphical cluster map based on Ripley’s K-function [39], pseudocolored according to relative fluorescence intensity in each individually detected particle. (TIF) [file pone.0100346.s011.tif]
